# ROBIN: A unified nanopore-based assay integrating intraoperative methylome classification and next-day comprehensive profiling for ultra-rapid tumor diagnosis

**DOI:** 10.1093/neuonc/noaf103

**Published:** 2025-05-20

**Authors:** Simon Deacon, Inswasti Cahyani, Nadine Holmes, Graeme Fox, Rory Munro, Satrio Wibowo, Thomas Murray, Hannah Mason, Mark Housley, Daniel Martin, Abdi Sharif, Areeba Patel, Robert Goldspring, Sebastian Brandner, Felix Sahm, Stuart Smith, Simon Paine, Matthew Loose

**Affiliations:** Nottingham University Hospitals NHS Trust, Nottingham, UK; School of Medicine, University of Nottingham, Nottingham, UK; School of Life Sciences, University of Nottingham, Nottingham, UK; School of Life Sciences, University of Nottingham, Nottingham, UK; School of Life Sciences, University of Nottingham, Nottingham, UK; School of Life Sciences, University of Nottingham, Nottingham, UK; School of Life Sciences, University of Nottingham, Nottingham, UK; School of Life Sciences, University of Nottingham, Nottingham, UK; Nottingham University Hospitals NHS Trust, Nottingham, UK; Nottingham University Hospitals NHS Trust, Nottingham, UK; Nottingham University Hospitals NHS Trust, Nottingham, UK; Nottingham University Hospitals NHS Trust, Nottingham, UK; Clinical Cooperation Unit Neuropathology, German Cancer Research Centre (DKFZ), Heidelberg, Germany; Nottingham University Hospitals NHS Trust, Nottingham, UK; Division of Neuropathology, National Hospital for Neurology and Neurosurgery, University College London Hospitals NHS Foundation Trust, London, UK; Department of Neuropathology, Heidelberg University Hospital, Heidelberg, Germany; Clinical Cooperation Unit Neuropathology, German Cancer Research Centre (DKFZ), Heidelberg, Germany; Nottingham University Hospitals NHS Trust, Nottingham, UK; School of Medicine, University of Nottingham, Nottingham, UK; Nottingham University Hospitals NHS Trust, Nottingham, UK; School of Medicine, University of Nottingham, Nottingham, UK; School of Life Sciences, University of Nottingham, Nottingham, UK

**Keywords:** diagnostics, glioma, intraoperative, methylation classification, nanopore

## Abstract

**Background:**

Advances in our technological capacity to interrogate CNS tumor biology have led to the ever increasing use of genomic sequencing in diagnostic decision making. Presently, CNS tumors are classified based on their epigenetic signatures, leading to a paradigm shift in diagnostic pathways. Such testing can be performed so rapidly using nanopore sequencing that results can be provided intraoperatively. This information greatly improves the fidelity of smear diagnosis and can help surgeons tailor their approach, balancing the risks of surgery with the likely benefit. Nevertheless, full integrated diagnosis may require subsequent additional assays to detect pathognomonic somatic mutations and structural variants, thereby delaying the time to final diagnosis.

**Methods:**

Here, we present ROBIN, a tool based on PromethION nanopore sequencing technology that can provide both real-time, intraoperative methylome classification and next-day comprehensive molecular profiling within a single assay. ROBIN utilizes 3 methylation classifiers to improve diagnostic performance in the intraoperative setting.

**Results:**

We demonstrate classifier performance on 50 prospective intraoperative cases, achieving a diagnostic turnaround time under 2 hours and generating robust tumor classifications within minutes of sequencing. Furthermore, ROBIN can detect single nucleotide variants, copy number variants, and structural variants in real time, and is able to inform a complete integrated diagnosis within 24 hours. Classifier performance demonstrated concordance with final integrated diagnosis in 90% of prospective cases.

**Conclusion:**

Nanopore sequencing can greatly improve turnaround times for standard-of-care diagnostic testing and is furthermore able to reliably provide clinically actionable intraoperative tumor classification.

**Key Points**

- A single assay can provide intraoperative methylome classification and next-day comprehensive molecular profiling, including single nucleotide variants, copy number variants, and structural variants.
- In 50 prospective intraoperative cases, concordance with standard of care was achieved in 90% of cases.

**Importance of the Study**

A key challenge for intraoperative tumor classification is balancing the competing needs of rapid sample processing and the requirement to generate DNA of adequate yield and read length for the subsequent detection of additional molecular features. Previous implementations of intraoperative, ultra-fast classification do not generate libraries of sufficient read length and pore occupancy for detecting additional molecular features beyond methylation-based classification alone. Implementations of nanopore sequencing for such comprehensive diagnostics have previously relied on ligation-based approaches that are not suitable for intraoperative use due to their longer turnaround time. To our knowledge, our study is the first to demonstrate an ultra-fast workflow which can integrate intraoperative classification and next-day full molecular profiling within a single assay. Furthermore, our study builds upon previous work by integrating 3 analytic tools to generate a robust intraoperative classification and improved diagnostic confidence, in contrast to previous works which rely on a single classification alone.

The 2021 WHO classification of CNS tumors substantially increased the mandated use of molecular testing in the diagnosis of CNS tumors. In particular, DNA methylation-based classification is now an essential criterion in the routine diagnosis of many tumor subtypes.^1^ DNA methylation of cytosines is maintained across cell divisions and is a means by which histogenesis can be traced to the cellular origin, despite neoplastic dedifferentiation.^2,3^ Currently, testing is implemented by microarray-based methods, such as Illumina EPIC arrays, which, because of high capital costs, are restricted to specialist tertiary care centers where high throughput can reduce per-assay costs.^4^ This requires inter-regional sample transport and multiplexed batch processing, which can cause diagnostic delays, with average turnaround times between several days to weeks.^4^

In contrast to array-based technologies and unraveling of a comprehensive molecular diagnosis by multiple assays (such as methylome array, DNA next-generation sequencing [NGS], and RNA NGS), nanopore sequencing (Oxford Nanopore Technologies) enables assessment of copy number profile and mutational and methylation analysis in a single assay via native strand, long-read sequencing. Capital and consumable costs are relatively low, and analysis can be performed on individual samples in real time. Nanopore sequencing has been shown to classify CNS tumors confidently on the basis of methylation status.^5,6^ Crucially, this technology enables affordable local sequencing with much faster turnaround times compared to the current standard of care (SoC).^7–9^

Current implementations of nanopore-based classification typically rely on low-coverage approaches using MinION flow cells, sequencing a small fraction of the genome’s total CpG sites. At such an ultra-low read depth, only binary methylation information can be obtained at most sites, and specialized tools have been developed to classify accurately based on this sparse methylation data.^8^ However, these tools are limited and are not able to report additional data, such as single nucleotide variants (SNVs) and structural variants (SVs), with high confidence due to low coverages. Nevertheless, capturing such genetic alterations is required to form a CNS WHO integrated diagnosis, and so additional assays such as RNA and DNA sequencing are required.

Here, we present an improved analysis pipeline performing integrated methylation-based tumor classification, copy number variation (CNV) profiling, SV calling, *MGMT* promoter methylation, and mutation detection. We use high-coverage PromethION flow cells and a modified transposase-based library preparation. We demonstrate the feasibility of using this technique for both intraoperative tumor classification and next-day final molecular diagnosis.

## Methods

### Patient Selection

Thirty primary CNS tumors with adequate available frozen tissue were selected for retrospective sequencing as per previously published protocols (Supplementary Materials).^5^ For this prospective cohort, patients were selected for nanopore-based sequencing if anticipated to have a primary intracranial neoplasm and be eligible for SoC methylation array. Consent for nanopore sequencing was obtained by the neurosurgical team preoperatively. However, 4 patients included in the study did not warrant further molecular testing by SoC approaches: 1 WHO Grade 1 meningioma with typical meningothelial morphology and no concerning features; 2 glioblastomas with no *IDH1 (R132H)* mutation by immunohistochemistry in patients over the age of 60 years; and a germinoma with diagnostic immunohistochemistry. As such, for these cases, the nanopore classifications were compared against the final CNS WHO Integrated Diagnosis alone, although nanopore sequencing nevertheless provided rapid evaluation of *MGMT* promoter methylation in the cases of glioblastoma. One case was excluded from the study during intraoperative DNA extraction due to the smear demonstrating metastatic adenocarcinoma.

### Retrospective Cohort DNA Extraction, Library Preparation, and Nanopore Sequencing

Sequencing of the retrospective cohort mirrored previously published protocols.^5^ DNA from frozen tumor samples were extracted using the Promega Maxwell® RSC Blood DNA Kit on a Promega Maxwell® RSC Instrument as per the methodology stated in the RapidCNS2 supplementary methods.^5^ Samples were quantified using a NanoDrop Spectrophotometer and an Invitrogen Qubit 3 Fluorometer High Sensitivity (HS) assay. DNA integrity and fragment-length profiles were assessed using the Agilent TapeStation ScreenTape Assay. Each genomic DNA sample was sheared using a Covaris g-TUBE at 4,200 rpm. Sequencing libraries were prepared from ~2 µg of sheared DNA, using the Ligation sequencing DNA V14 protocol as per manufacturer’s instructions (ONT SQK-LSK114). Each library was run over one MinION flow cell (ONT FLO-MIN114 R10.4.1) on a GridION X5 sequencer. To maximize sequencing output, each flow cell was flushed and reloaded every 24 hours over a 3-day total sequencing runtime, using the Flow Cell Wash Kit (ONT WSH004).

### Intraoperative DNA Extraction

Tissue was collected fresh, direct from the operating theater, and 3 spatially distant tumor samples were selected macroscopically for further processing. All tissue selected was in excess of that required for SoC neuropathological workup. For each sample, between 5 and 25 mg tissue was taken for DNA extraction, and adjacent tissue was reserved for intraoperative smear analysis. A minimum input tissue weight of 5 mg enables extraction on stereotactic biopsy samples. DNA extraction was performed using the QIAmp Fast DNA Tissue Kit, as per the manufacturer’s instructions. During the DNA extraction, the neuropathologist reported in parallel on the smear preparation and advised which of the 3 samples was most suitable for sequencing, for example excluding samples with extensive gross necrosis or a markedly low tumor cell fraction. Quantitative estimation of tumor fraction is difficult, if not impossible, on smear preparation alone and expert neuropathologist opinion determined tissue suitability for sequencing. While there is a degree of interobserver variability in such estimation, frozen sectioning and quantitative tumor estimation cannot be performed within the intraoperative timeframe. All DNA extraction reagents are stable at room temperature, avoiding the need for specialist storage and enabling the preparation of reagents prior to the operation, allowing immediate processing upon sample receipt.

### Intraoperative Library Preparation and Sequencing

Samples were prepared with the ONT ultra-long kit using an adjusted protocol (dx.doi.org/10.17504/protocols.io.bp2l6xepklqe/v1). DNA samples were diluted in nuclease-free water, and concentrations were measured using the Qubit 3 Fluorometer (Thermo Fisher Scientific). Each DNA sample was sheared using 18 passes of a 30G needle, producing fragments with a length peak in the region of 25 kbp. Sheared DNA (600 ng) was tagmented in 3 μL FRA at 30 °C for 5 minutes, followed by the addition of sequencing adapters in 1 μL Rapid Adapter. The library was then purified using 100 μL AmpureXP DNA binding beads (Beckman Coulter; A63882). Library concentration was again measured prior to flow cell loading using the Invitrogen™ Qubit™ 3 Fluorometer and High Sensitivity assay. The library was loaded onto R10.4.1 PromethION flow cells. Sequencing was commenced 5 minutes after library loading and performed for up to 24 hours. Real-time basecalling was performed using MinKNOW; reads were called using the high-accuracy model with 5mC and 5hmC modifications. The Oxford Nanopore Technologies tool “modkit” with “adjust-mods --convert h m” was used to merge the 5hmC and 5mC data by converting 5hmC tags to 5mC tags and summing the probabilities together into a single value. Reads were mapped to GRCh38, and the resulting BAM files were used for subsequent analysis. Adaptive, targeted sequencing of the regions-of-interest (ROIs) was performed using the Readfish tool^10^ and a BED file of targets. This included 3,487 targets, including every chromosome other than Y as described in Patel et al.^5^ Basecalling for adaptive sampling was carried out on a dedicated Nvidia 4090 GPU running the dorado basecaller using the appropriate FAST mode model and mapping to hg38 with alt chromosomes removed.

### Real-Time Intraoperative Visualization and Reporting

To facilitate real-time analysis of data, we have developed ROBIN (Rapid nanpOre Brain intraoperatIve classificatioN). ROBIN provides a graphical interface to present sequencing results as data are being generated (https://github.com/looselab/robin). ROBIN integrates multiple classifiers of methylation, CNV changes, coverage over targets and SNVs, and *MGMT* promoter methylation and indicates candidate fusions during sequencing in one graphical user interface. The methylation classifiers used were: Sturgeon, CrossNN, and the random forest (RF) approach from RapidCNS2.^5,8,11^ The threshold value representing a confident classification score is particular to each classifier and is defined as follows, based on thresholds proposed in the corresponding original publications: ≥70 (RF); ≥0.8 (sturgeon); ≥0.2 (CrossNN). Copy number changes are dynamically visualized using CNV from BAM, but manual visual inspection is required for confirmation (https://github.com/adoni5/cnv_from_bam). SNVs are analyzed at a minimum read depth of 10× using ClairS-TO; the threshold defining the required number of supporting reads to call an event is described in their documentation (https://github.com/HKU-BAL/ClairS-TO). Resultant SNVs are annotated using snpEff and snpSift; only mutations with previously reported clinical significance are supported.^12,13^ All SNV calls were manually verified by visualization in real time through IGV reports embedded in the ROBIN interface (https://github.com/igvteam/igv-reports). *MGMT* methylation status is analyzed as in RapidCNS2 and visualized using methylartist.^5,14^ This prediction of *MGMT* methylation status uses 137 CpG sites as per Patel et al., which differs from the model used for array-based *MGMT* prediction.^5^ Methylation classification, CNV profiling, and SNV calling were internally validated using a separate nextflow-based implementation of ROBIN (https://github.com/graemefox/SCARLET). Candidate gene fusions are identified by inspection of long reads, either aligning to 2 disparate targets in the adaptive sampling BED file, or aligning to both one gene from the BED file and one target elsewhere in the genome. Fusions are called where there are at least 3 supporting reads identified, and visualized using https://github.com/Edinburgh-Genome-Foundry/DnaFeaturesViewer.

ROBIN operates through a web interface, although running on the local computer performing the sequencing. As a consequence, the output of ROBIN can be viewed by a pathologist remote to the sequencing computer, enabling a confident diagnosis to be made by a clinician in real time during sequencing and permitting geographically separate sequencing and clinical analysis. While the results of nanopore-based tumor sequencing and classification were available prior to the completion of SoC testing, this information was treated strictly as research use only, and no data were used to inform patient care.

## Classifier Versions

Three major brain tumor classifiers have been published and are used in the present study: the RF-based classifier NanoDx,^5,6^ its subsequent neural network-based implementation, CrossNN,^11^ and the Sturgeon neural network-based classifier.^8^ The Sturgeon classifier is specifically optimized to handle the sparse data typically generated by nanopore sequencing, having been trained using simulated cases generated from subsampling methylation array data. On the other hand, CrossNN aims to be a platform-agnostic classifier that can handle both array- and nanopore-derived methylation data. Crucially, all 3 classifiers are publicly available and are trained using open-access data generated during the development of the antecedent array-based classification.^3^ However, it is important to note that the Heidelberg dataset has undergone multiple and substantial subsequent iterations using additional patient data since this initial publication. Newer versions of this reference dataset define novel entities that were not included in earlier classifier versions, and many of these entities are predicated solely on their unique methylation signatures (https://www.molecularneuropathology.org/mnp/classifiers). As such, these nanopore-based classifiers are a priori limited in their ability to correctly diagnose these tumor subtypes. Furthermore, methylation data have been shown to have clinical utility in the prognostic stratification of meningiomas, yet such features are absent from the earlier reference data upon which the nanopore classifiers were trained.^15,16^

## Results

### Same-Day Methylation-Based CNS Tumor Classification

**Figure 1.**
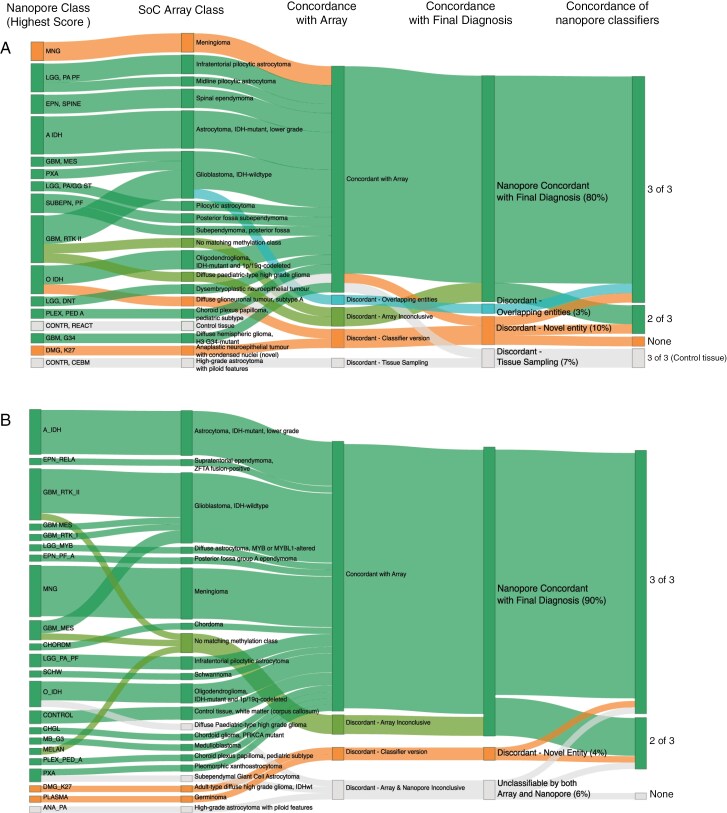
Nanopore classifications vs SoC testing. A) Retrospective cohort. B) Prospective (intraoperative) cohort. The highest-scoring nanopore classification after 24 h is displayed, with the exception of one chordoma case that was correctly classified intraoperatively yet classified as control tissue after 24 h. Concordance with array was determined irrespective of glioblastoma subclass.

Nanopore sequencing enables rapid methylation-based tumor classification. Thirty frozen samples were retrospectively sequenced using MinION flow cells to validate the methodology, and 50 tumors were subsequently prospectively sequenced intraoperatively using PromethION flow cells. Of these, 24 (80%) and 45 (90%) of the respective cohorts were correctly classified by nanopore after 24 hours total sequencing time (Figure 1). Classification results were validated against both SoC methylation array and final integrated diagnosis (Supplementary Tables 1 and 2). The major causes of misclassification were common across both cohorts (Supplementary Tables 3 and 4), including misclassifications of novel entities absent from the nanopore classifiers and low tumoral DNA in sample tissue. In neither the retrospective nor the prospective cohorts, did the ONT-based pipeline deliver an erroneous result, the reasons for which could not be understood or accounted for by either sample bias or differences in classifier versions.

### Next-Day Comprehensive Molecular Profiling of CNS Tumors

**Figure 2.**
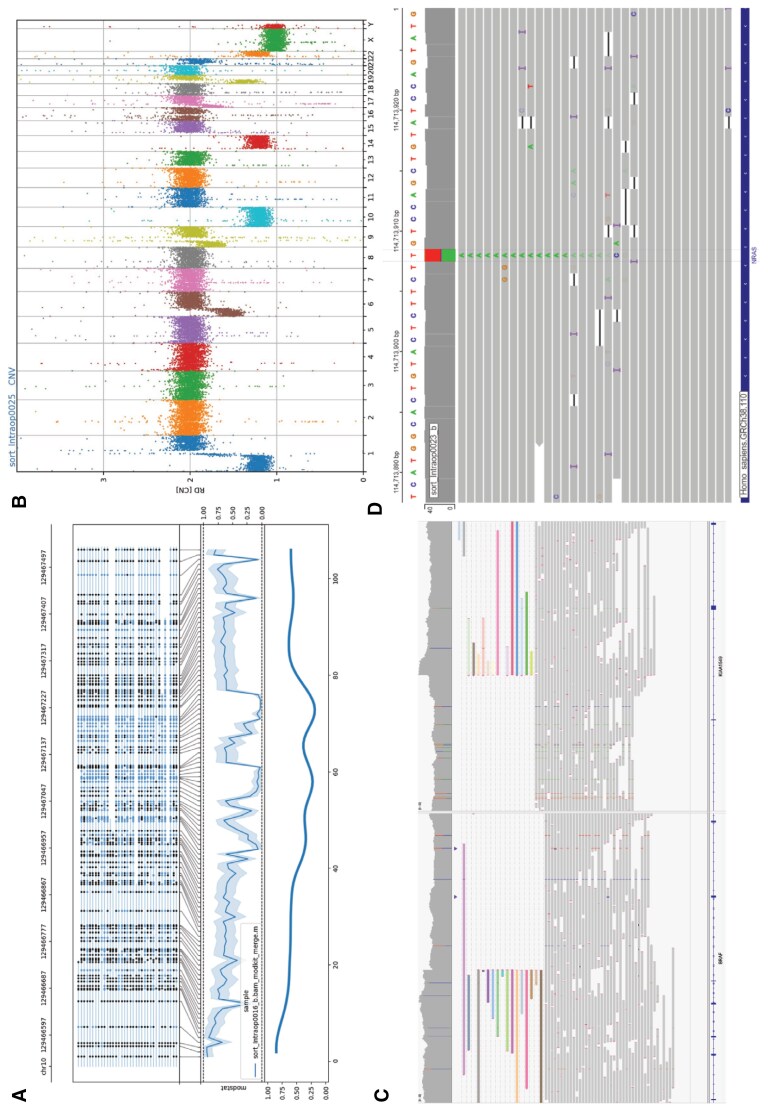
Additional molecular features captured within the next-day report. A) Methylation distribution at the *MGMT* promoter in a glioblastoma. Top panel: each line corresponds to a single read; black dots indicate methylated sites, and blue dots indicate nonmethylated sites. Middle panel: average methylation score across the *MGMT* promoter. Bottom panel: smoothed average. Clinically relevant methylation is defined as >25% methylation across the region. B) Copy number plot of an oligodendroglioma, demonstrating the canonical codeletion of 1p and 19q chromosome arms. C) Demonstration of a pathognomonic *BRAF::KIAA1549* fusion in a pilocytic astrocytoma. D) Demonstration of a c.A182T (p.Q61L) mutation of *NRAS* in a case of metastatic melanoma.

Alongside methylation-based classification, other molecular features are crucial for accurate diagnosis. For example, CNV changes are part of some CNS WHO diagnostic criteria (Figure 2).^1^ Since both on-target and off-target reads contribute to the CNV data, these plots can be rapidly and intraoperatively generated by ROBIN, with diagnostic evidence for CNV changes apparent even at low coverages. However, visual inspection of the copy number plots by the reporting neuropathologist is required, as is customary practice for many CNV reporting tools. Copy number profiles generated by ROBIN were in concordance with array-generated data. For example, most cases of glioblastoma demonstrated the canonical gain of chromosome 7 and loss of chromosome 10. All cases of oligodendroglioma exhibited the pathognomonic loss of chromosomes 1p and 19q, including one case of anaplastic oligodendroglioma (CNS WHO Grade 3) exhibiting additional noncanonical deletions of multiple chromosome arms (Supplementary Figure 1). Additionally, intrachromosomal events such as codeletion of CDKN2A/B in astrocytoma are reliably detected (Supplementary Figure 2).

A key strength of adaptive sampling coupled with PromethION flow cells is that much higher coverage can be generated, within a shorter timeframe when compared to standard MinION-based approaches. This enabled confident identification of relevant SNVs and SVs in our cohort, evaluated against known tumor hallmarks and, where available, SoC testing. *IDH1/*2 mutations were correctly identified in 3 of 3 oligodendrogliomas and 6 of 7 astrocytomas (Supplementary Table 5); in one case, low tumor DNA fraction impaired analysis. Additional known SNVs, such as *TERT* promoter mutations in glioblastoma and *TP53* mutations in astrocytoma, were reliably identified. One case was reliably classified as a chordoid glioma, which has been reported to harbor the p.D463H (c.1387G > C) missense mutation in the *PRKCA* gene. In our analysis, this SNV was not automatically called within the report; however, manual inspection of the data confirmed the SNV in 9 of 35 reads. As such, SNV calling by ROBIN is limited to high-confidence calls at relatively high variant allele frequencies, and ancillary panel testing may be required in cases where tumor fraction is low.

Identification of specific SVs is critical in the diagnosis of certain tumors, and the distinct advantage of nanopore sequencing is demonstrated by the identification of novel, complex fusions via long reads spanning the fusion event. In our cohort, 5 cases demonstrated clinically relevant fusions (Supplementary Figure 3). Two cases demonstrated novel, complex fusions. “MYB- or MYBL1-altered diffuse astrocytoma” is a rare entity defined by a canonical fusion event of *MYB-* or *MYB1L*, with the most common partner genes *PCDHGA1*, *MMP16*, and *MAML2*. However, in our cohort, we report a case with a fusion involving MYB and the partner genes *EYA4* and *HBS1L.* Secondly, we report an intracranial schwannoma with a novel complex *VGLL3::SAM13::CHD7* fusion. While consistent with the known *VGLL3::CHD7* fusion demonstrated by this tumor, the additional partner of *SAM13* has not previously been reported.^17^ A further 2 cases demonstrated pathognomonic fusion events: *KIA1549::BRAF* fusion in a pilocytic astrocytoma, and *ZFTA::RELA* fusion in a supratentorial ependymoma. While the majority of glioblastomas do not harbor fusions, one case within our cohort demonstrated a *SEPTIN14::EGFR* fusion, the commonest fusion demonstrated in glioblastoma.^18^ A singular case did not demonstrate a canonical fusion: a pilocytic astrocytoma arising in the context of germline *NF1* mutation. No case reliably classified by methylome analysis as an entity harboring a pathognomonic fusion without corresponding long reads supporting the fusion event.

The methylation level of the *MGMT* promoter is a clinically important biomarker, identifying patients who are likely to respond to adjuvant temozolomide chemotherapy. Nanopore-derived *MGMT* status was concordant with array-based predicted *MGMT* status in 44 of 46 cases in the prospective cohort (Supplementary Table 6), and 25 of 29 cases in the retrospective cohort. One discrepant case had a methylation score very close to the cutoff, suggesting potentially a subtle difference in methylation thresholds between array and nanopore approaches. Indeed, such equivocal low-level methylation of *MGMT* may still be an indicator of potential sensitivity to temozolomide treatment.^19^

### Intraoperative Methylation-Based Classification

**Figure 3.**
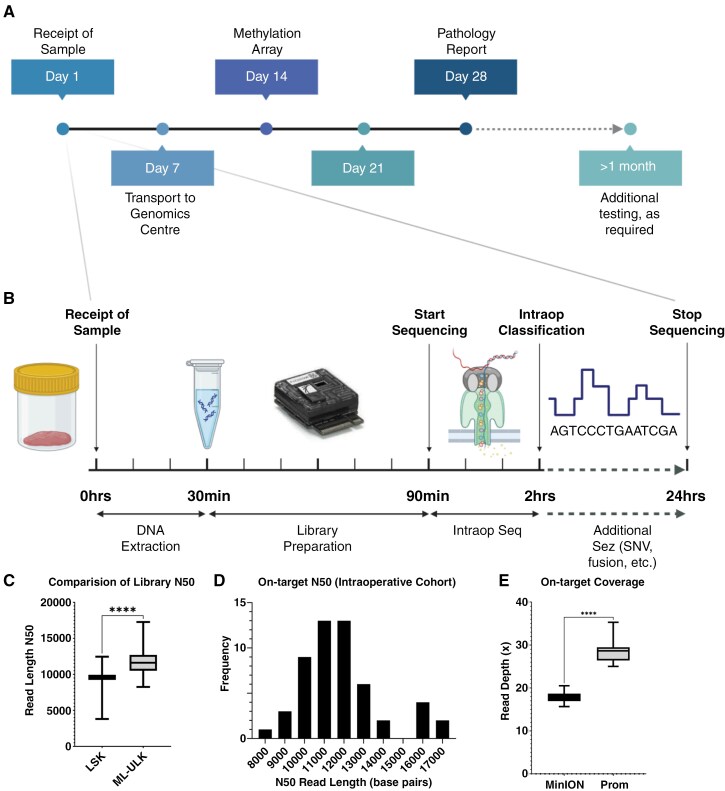
Intraoperative library preparation and sequencing workflow. A) Typical timeline of SoC testing. B) Exemplar intraoperative DNA extraction and sequencing workflow (Images genrated from BioRender.com). C) Average library N50 of the intraoperative protocol improved upon that of the retrospective cohort. D) Histogram of on-target N50 values for the intraoperative cohort (mean N50 = 11,739 bp). E) On-target coverage was significantly superior in the intraoperative cohort cases (PromethION; runtime 24 h) compared to the retrospective validation cohort (MinION; runtime 72 h).

A key challenge for intraoperative tumor classification is balancing the competing needs of rapid sample processing and the requirement to generate DNA of adequate yield and read length for the subsequent detection of additional molecular features. Previous implementations of nanopore-based comprehensive diagnosis, beyond methylation classification alone, have used ligation approaches and are not suitable for intraoperative use due to their longer turnaround time.^5^ Similarly, while our retrospective cohort generated libraries of sufficient quality, overnight tissue lysis and a ligation-based library inordinately delayed time to diagnosis. Our prospective library protocol utilized an adapted transposase-based approach that enabled rapid sample preparation within 90 minutes of tissue receipt while preserving the optimal DNA read length for adaptive sampling (Figure 3A and B).

Conversely, ultra-fast approaches that require highly disruptive mechanical lysis steps and obviate the need for DNA purification do not generate libraries of sufficient read length and pore occupancy for detecting additional molecular features beyond methylation-based classification.^8^ Indeed, while these authors opted not to utilize adaptive sampling in the intraoperative setting, it is precisely these additional molecular features, such as SNV and fusion-calling, which most benefit from such enrichment, if used alongside libraries of appropriate quality. In our data, our intraoperative protocol produced longer read length libraries compared to the retrospective cohort, which followed a previously published ligation-based protocol using g-TUBE shearing^5^ (Figure 3C). For the intraoperative cohort, we achieve a mean N50 of 11,739 bp, compared to 9,195 bp of the ligation-based protocol (*P* < 0.0001) (Figure 3D), and a significantly higher on-target coverage using PromethION flowcells (Figure 3E).

**Figure 4.**
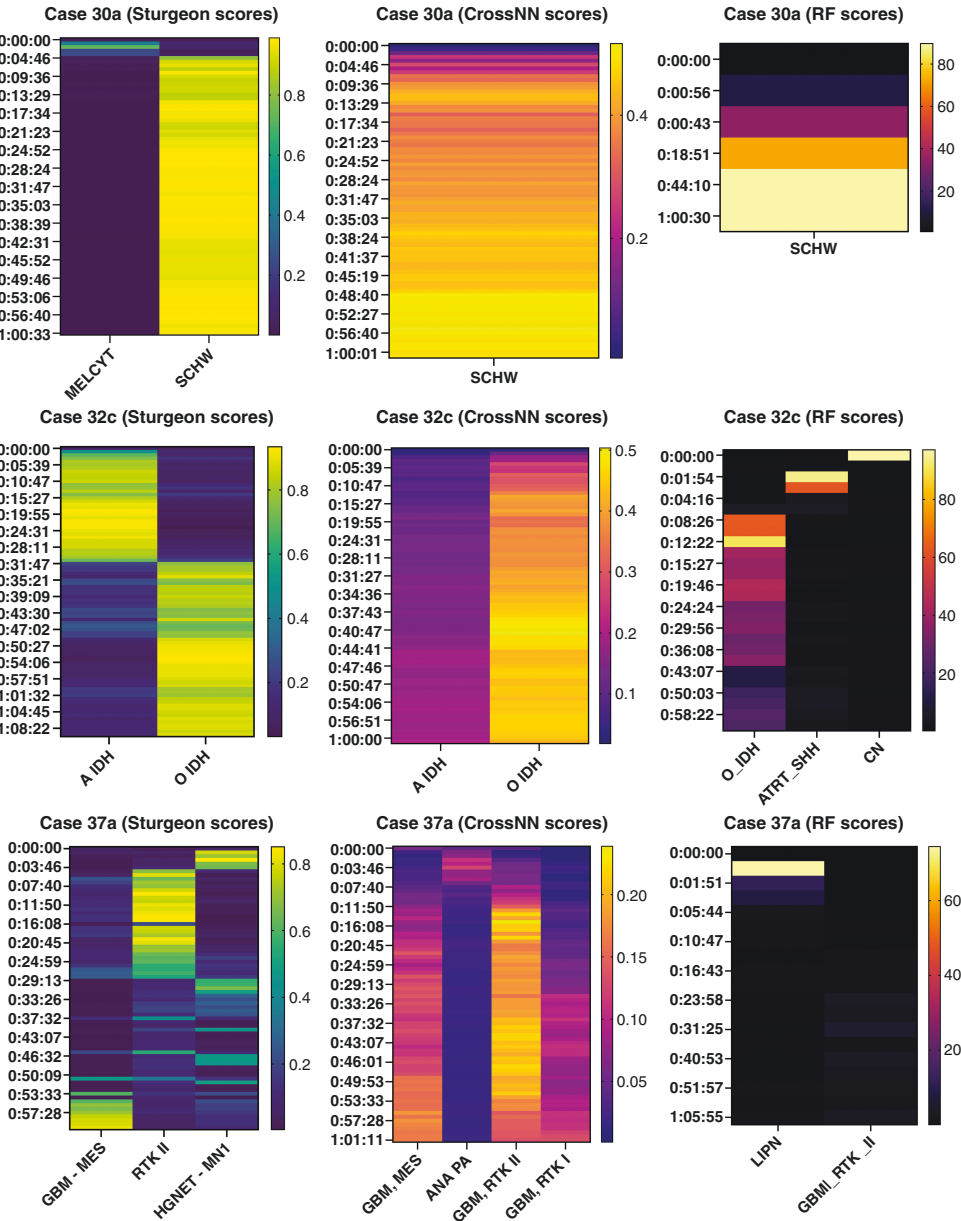
Classifiers confidently predict tumor type during intraoperative sequencing. A) Case example of intracerebral schwannoma which was confidently classified on all 3 classifiers. B) Example of a case of oligodendroglioma in which there was some heterogeneity between- and within-classifiers during intraoperative sequencing, but ultimately all classifiers reached concordance. C) Example of a case in which one classifier failed to confidently predict the tumor subtype, yet the remaining 2 classifiers were successful.

Thirty-eight cases (76%) within the prospective cohort were classified intraoperatively, defined as a confident classification by 2 or more classifiers within 1 hour of sequencing. Classifiers demonstrated variable performance intraoperatively (Supplementary Figure 4). Both neural network-based classifiers (Sturgeon and CrossNN) were able to confidently classify cases within minutes, whereas RF performance was slower (Figure 4A). We hypothesize that, in a subset of cases, classifier performance was poor during early intraoperative sequencing, due to the sparsity of the data and overall low coverage of CpG sites (Supplementary Figure 5). The Sturgeon classifier demonstrated a tendency to overconfidently misclassify cases, dynamically switching class mid-sequencing (Figure 4B). As such, multiple classifiers using different methodologies are particularly important during intraoperative classification where congruency of classification can support confident clinical interpretation, or where a single tool fails to classify (Figure 4C).

## Discussion

We present a protocol that can generate a comprehensive molecular diagnosis within an improved timeframe compared with current SoC diagnosis. In our prospective cohort, the mean turnaround time for SoC methylation-based classification was 33 days (range: 19–81 days). This turnaround time encompasses both delays locally, such as neuropathological assessment and immunohistochemical studies prior to referral (mean: 12 days), and delays following receipt of the sample at the specialist laboratory (mean: 21 days). While local delays may be mitigated by routinely requesting methylation array analysis prior to initial neuropathological workup, such an approach may limit the selection of optimal tissue for molecular studies and risks costly molecular testing in cases where it is either unsuitable or not required for diagnosis. In the subset of cases where further molecular testing such as RNA- and DNA-based NGS was required for SNV and fusion-calling, the mean turnaround time was 68 days (range: 54–103 days).

The equivalent mean turnaround times for our ultra-fast pipeline were within 24 hours for final comprehensive diagnosis of the 90% of cases which were classifiable. In many cases, methylation classification and identification of relevant SNVs and CNVs were achieved within a few hours of sequencing, but precise quantification of time-to-result is complex. Implementing nanopore-based sequencing for routine practice would democratize access to rapid results, with immediate benefits to patients, medical professionals, and the wider health system. Nanopore-based intraoperative classification enables characterization of the tumor subtype in far greater detail than smear or frozen section alone. While expert neuropathological examination of the smear is the cornerstone of intraoperative diagnosis, it can never achieve either the degree of nuance or confidence of diagnosis offered by sequencing; not least because many tumor entities are now explicitly defined by their molecular hallmarks in the absence of defining morphological features.

This study is from a single UK center, and our prospective cohort illustrates the complexity of neuropathological diagnoses and the potential impact of this technology on clinical practice. For example, many pediatric tumor types present as nonspecific, primitive cells on intraoperative smear and have a wide differential diagnosis. In one pediatric case, the surgical decision was made for subtotal resection while awaiting full molecular diagnostic workup. The child subsequently required repeat surgery once SoC testing had been performed, and the tumor was diagnosed as a *RELA::ZFTA*-fused ependymoma 19 days postoperatively. In contrast, nanopore-based analysis correctly classified the tumor intraoperatively and could have spared the patient unnecessary surgical risk. Rare tumors are also often diagnostically challenging, and this may delay the initiation of appropriate treatment or risk overtreatment. In our cohort, we rapidly classified an intracranial schwannoma within minutes of sequencing and demonstrated a novel pathognomonic VGLL3 fusion within 24 hours. Preoperative imaging and clinical assessment were suggestive of an IDH-mutant astrocytoma. Astrocytomas are CNS WHO Grades 2 or 3 and confer a lifelong risk of recurrence and high-grade transformation, whereas schwannoma is CNS WHO Grade 1 and surgery is curative. In this context, rapid nanopore diagnosis could have spared the patient weeks of anxiety and distress waiting for a comprehensive diagnosis. Another major strength of nanopore-based classification is its minimal input tissue requirement, making classification even on the basis of stereotactic biopsies feasible. However, it must be stressed that, under the ethics in which this study was conducted, clinical teams were not made aware of the results of sequencing. As such, comprehensive assessment of the clinical impact of such rapid diagnosis is beyond the scope of this paper and will need to be addressed under the auspices of a clinical trial, with full ethical approval to action this data clinically.

Nanopore technology offers multiple advantages; however, neuropathological interpretation and integration of the data remains crucial. During sequencing, the pathologist can situate the real-time results within the context of the smear examination and the broader clinical and radiological differential diagnosis, ensuring that methylation classification is congruent with these data. For example, some cases which classified as control tissue by one classifier were successfully classified as astrocytoma on the remaining 2 classifiers. In addition, copy number changes may further aid diagnosis in cases where classification is uncertain: early glioblastoma or the sampling of the infiltrative zone, for example, may misclassify as control tissue but can nevertheless demonstrate the chromosome 7 gain and chromosome 10 loss alongside the concomitant *EGFR* amplification characteristic of glioblastoma. However, caution must be taken in interpreting such results; a classification of tumor in 2 of 3 classifiers may be accepted by the neuropathologist if, for example, these findings are consistent with the intraoperative smear. Conversely, in our cohort, nanopore sequencing was able to correctly classify a case of melanoma that was not recognized as such on intraoperative smear. If in keeping with the clinical context, such data may prompt the neuropathologist to re-examine the smear or to request more tissue in order to perform a second intraoperative smear or expedite adjuvant testing. As analysis is rapid, if the pipeline does not produce diagnostically useful data and additional material is available, DNA can be extracted from a different region and analyzed with only a minimal delay.

A definitive, quantitative threshold for classification cannot currently be defined. Furthermore, it is not possible at this time to codify the different classifiers to give a single common score. The classifier scores must be considered within the clinical context, and the concordance between multiple classifiers may generate stronger evidence of a robust classification. In our data, using multiple classifiers is vital to inform intraoperative interpretation, thus reducing the risk of a misleading classification based on a single output while potentially enabling a more definitive diagnosis in indeterminate cases. Similarly, the clinical relevance of CNV changes is highly variable, and the neuropathologist is again vital to the accurate interpretation of these changes on a case-by-case basis. For example, all *IDH*-mutant astrocytomas in our cohort were classified as low grade by methylation array, both by nanopore sequencing and SoC array. Despite this, 4 astrocytomas exhibited codeletion of *CDKN2A/B* on CNV profiling, thus carrying a far worse prognosis (CNS WHO Grade 4) than the methylation class alone would indicate (likely CNS WHO Grade 2). Furthermore, the smear preparation in these cases did not reveal any high-grade morphological features, and thus intraoperative identification of the high-grade copy number changes may have altered the surgical approach. Additional information on combined CNV load in astrocytoma can be readily integrated into the analysis and further aid the grading and prognostication of IDH-mutant astrocytomas.^20^ As confidence in these approaches grows, it is likely that traditional morphological methods, such as immunohistochemistry, will become redundant, at least for a subset of cases. This would further augment the economic argument for a nanopore-based pipeline over the current SoC pathway.

Detection of pathogenic SNVs remains a vital aspect of comprehensive molecular diagnosis. While we demonstrate that next-day detection of SNVs is feasible, it is acknowledged that panel-based sequencing techniques can achieve much higher coverage of target genes. We achieved an average on-target coverage of approximately 30× using PromethION flow cells, which is improved from the coverage that we achieved with ligation-based MinION approaches. Nevertheless, a minimum coverage of 250× or greater has been recommended for somatic variant detection, and far greater coverage is suggested for detection of low frequency variants.^21^ We propose that while nanopore-based SNV detection is a helpful additional data point when securing an integrated diagnosis, it is an adjunct to, and an indication for, conventional panel-based confirmatory sequencing at present and cannot be used, yet, for novel mutation discovery. This is likely to change as further improvements in library preparation, adaptive sampling technology, and flow cell performance may offer further gains in on-target coverage.

A common cause of misclassification using nanopore data is due to differences in classifier versions; current nanopore tools have been trained primarily on the original Capper dataset.^3^ A subset of our novel cases was classified using a platform-agnostic tool trained using the latest version of the DKFZ classifier (version 12.8) and were able to return a correct diagnosis.^22^ This should prompt the clinical and scientific communities to pool data to generate an intraoperative nanopore classification tool which can incorporate novel entities in the same manner as the current SoC array-based classification tools.

The pipeline that we describe is low cost and readily adoptable with a small laboratory footprint. We estimate that, inclusive of washing and reusing flowcells, a single sample can be analyzed for approximately £400. Such a figure is comparable to the cost of methylation array and becomes more cost effective by sparing the need for ancillary testing such as NGS. Furthermore, while the cost of the promethION device is more expensive per-sample than alternative approaches implemented with minION, we can achieve a final diagnosis in 24 hours on a single library. In contrast, minION sequencing requires 72 hours runtime and labor-intensive serial loading of libraries to achieve a similar end point. The requirement of fresh tissue may limit adoption of the technique in centers where this is not practicable, for example due to colocality with neurosurgical theaters and access to adequate dissection and freezer facilities. However, collection of fresh tissue is increasingly a reasonable expectation of a diagnostic neuropathology laboratory meeting current WHO diagnostic standards. We anticipate that such barriers to implementation will diminish over time, particularly as the use of preservatives such as RNAlater becomes more widespread, which mitigate many of these problems.

## Conclusion

In conclusion, we demonstrate a novel diagnostic assay which can provide reliable methylation-based tumor classification within a 2-hour intraoperative timeframe, and complete diagnostic molecular profiling, including SVs and SNVs, within 24 hours. We anticipate that this technology has the potential to radically alter the present SoC for CNS tumor diagnosis. Therefore, it is vital that concurrent efforts are made to ensure that sufficient healthcare professionals are adequately trained in the analysis of this complex data. The role of the neuropathologist is evolving, as they will be called upon to interpret and decipher multifaceted data in real time and integrate it with the morphological, clinical, and radiological findings. Only by situating this data within the unique clinical context of the patient will better outcomes be achieved, and efficacious tailored therapies be realizable.

For this technology to be best delivered for the benefit of patients, it is vital that pathways are designed around decentralized, near-patient sequencing. Capital costs of nanopore devices are low and the technique of sequencing can be easily adopted by nonexpert laboratories. To leverage maximal reduction in diagnostic turnaround times, only data should move between hospitals; expensive and slow transport of tissue must be reduced to a minimum. It must be acknowledged that a common cause of misclassification using nanopore data is due to differences in classifier versions; a subset of our novel cases were successfully classified using an updated platform-agnostic tool.^22^ This should prompt the clinical and scientific communities to pool data to generate an intraoperative nanopore classification tool which can incorporate novel entities in the same manner as the current SoC array-based classification tools. Efforts must be made to centralize data storage, to ensure reproducible, robust analysis of the data and enable the generation of a nanopore-specific reference cohort.

## Supplementary Material

noaf103_suppl_Supplementary_Tables_1-7_Figures_1-5

## Data Availability

De-identified individual participant data that underlie the results reported in this article will be made available upon reasonable request. The ROBIN code used for the analysis is available at https://github.com/looselab/robin.
