# Supplementary material for: ROBIN: A unified nanopore-based assay integrating intraoperative methylome classification and next-day comprehensive profiling for ultra-rapid tumor diagnosis
Source: Neuro Oncol. 2025 May 20;27(8):2035–46. doi: 10.1093/neuonc/noaf103 (PMC12448888; doi:10.1093/neuonc/noaf103)
Supplement: noaf103_suppl_Supplementary_Tables_1-7_Figures_1-5 [file noaf103_suppl_supplementary_tables_1-7_figures_1-5.docx]

Supplementary Appendix

ROBIN: A unified nanopore-based sequencing assay integrating real-time, intraoperative methylome classification and next-day comprehensive molecular brain tumour profiling for ultra-rapid tumour diagnostics

Deacon S^1,2^, Cahyani I^3^, Holmes N^3^, Fox G^3^, Munro R^3^, Wibowo S^3^, Murray T^3^, Mason H^2^, Housley M^2^, Martin D^2^, Sharif A^2^, Patel A^4^, Goldspring R^2^, Brandner S^5^, Sahm F^4,6^, Smith S^1,2^, Paine SML^1,2^, Loose M^3^

1 School of Medicine, University of Nottingham, Nottingham, United Kingdom

2 Nottingham University Hospitals NHS Trust, Nottingham, United Kingdom

3 School of Life Sciences, University of Nottingham, Nottingham, United Kingdom

4 Clinical Cooperation Unit Neuropathology, German Cancer Research Centre (DKFZ), Heidelberg, Germany

5 Division of Neuropathology, National Hospital for Neurology and Neurosurgery, University College London Hospitals NHS Foundation Trust, London, United Kingdom

6 Department of Neuropathology, Heidelberg University Hospital, Heidelberg, Germany

Correspondence: [matt.loose@nottingham.ac.uk](mailto:matt.loose@nottingham.ac.uk)

Table of Contents

1. Additional Methods
   1. Nextflow pipeline: integrated methylation classification, SNV and SV calling
2. Supplementary Figures
   1. Supplementary Data Figure 1: Copy number heatmaps
   2. Supplementary Data Figure 2: Copy number plot of all astrocytomas in the prospective cohort
   3. Supplementary Data Figure 3: Pathognomonic fusion events within the intraoperative cohort.
   4. Supplementary Data Figure 4: Intraoperative sequencing results over 1 hour
3. Supplementary Tables
   1. Supplementary Data Table 1: Overview of Intraoperative cases
   2. Supplementary Data Table 2: Overview of Retrospective cases
   3. Supplementary Data Table 3a: Discrepant cases in retrospective cohort
   4. Supplementary Data Table 3b: Novel cases in retrospective cohort
   5. Supplementary Data Table 4a: Discrepant cases in intraoperative cohort
   6. Supplementary Data Table 4b: Novel entities in intraoperative cohort
   7. Supplementary Data Table 5: Summary of selected additional diagnostic information
   8. Supplementary Data Table 6a: MGMT promoter methylation (intraoperative cohort)
   9. Supplementary Data Table 6b: MGMT promoter methylation (retrospective cohort)

**Nextflow pipeline: integrated methylation classification, SNV and SV calling**

The nextflow pipeline is publicly available at <https://github.com/graemefox/SCARLET>. Reads were aligned to the GRCh38 reference genome using minimap2 ^12^ generating BAM files. These sorted, indexed, BAM files were then used as inputs to a new Nextflow pipeline which performs a full molecular characterisation of the tumour sample. This pipeline incorporates several modules from the Oxford Nanopore Technologies ‘epi2me-labs’ wf-human-variation Nextflow workflow (v.2.3.0) which calls SNPs, structural variants (SV), copy number variations (CNV), aggregates modified base data, and generates reports. Briefly, the packages used for these analyses were Clair3 (v.1.0.8), sniffles2 (v.2.0.7), modkit (github.com/nanoporetech/modkit; v.0.3.0), and QDNASEQ (v.1.34.0)^3–5^. Aggregated modified base data, containing the methylation probabilities, are used to perform tumour classification using three methods: rapidCNS2, Sturgeon (v.0.4.4), and nanoDx (v1.0rc3). Variant calls generated by wf-human-variation (clairS) and those generated separately by clairS_To (v0.1.0), are annotated (ClinVar v.20240708, annovar v.2019-10-24) and reported in an interactive html alignment generated by igv_reports (v1.12.0), highlighting somatic mutations where possible. The methylation of the *MGMT* promoter region is analysed and reported using the method detailed in the rapidCNS2 pipeline. Variants and classifications are reported alongside depth of coverage estimates and a copy number variation plot in a final report (Figure 2). Briefly, the packages used for these analyses were CNVpytor (v.1.3.1), samtools (v.1.13), mosdepth (v.0.3.4), methylartist (v1.2.7), vcftools (v.0.1.16), bedtools (v.2.3.0), annovar with hg38 databases, and R scripts associated with the Rapid-CNS ^1^ pipeline (Quinlan and Hall, 2010; Wang, Li and Hakonarson, 2010; Danecek et al., 2011; Suvakov et al., 2021; R Core Team, 2022).


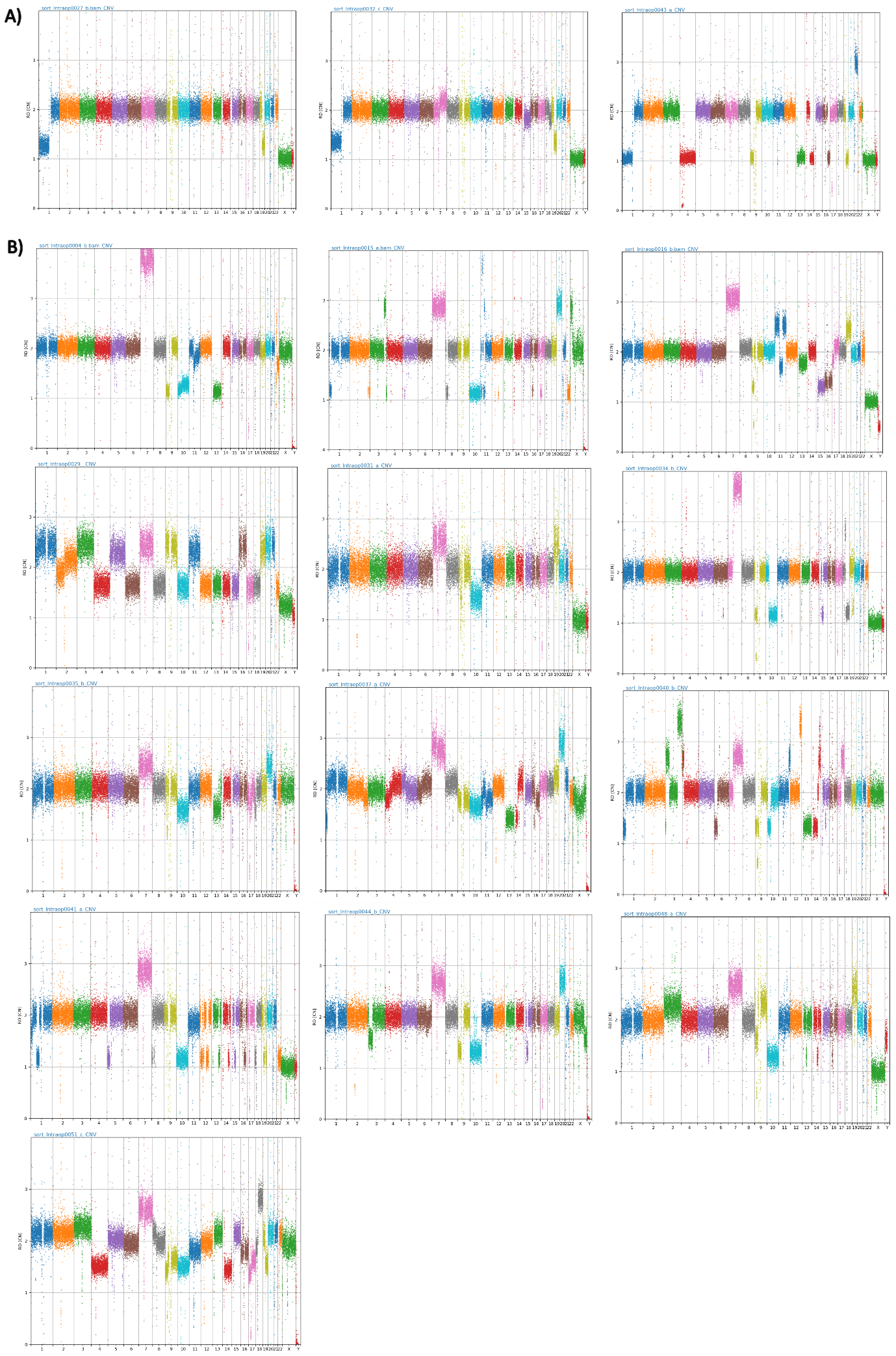


**Supplementary Data Figure 1: Copy number heatmaps** *A:* Oligodendroglioma, exhibiting codeletion of 1p and 19q. Note, case 43 is an atypical higher grade oligodendroglioma, exhibiting multiple additional copy number changes including loss of CKDN2A/B locus on chromosome 9; *B:* Glioblastoma, demonstrating the canonical gain of chromosome 7 and loss of chromosome 10 in many cases, alongside additional, complex copy number changes.

**
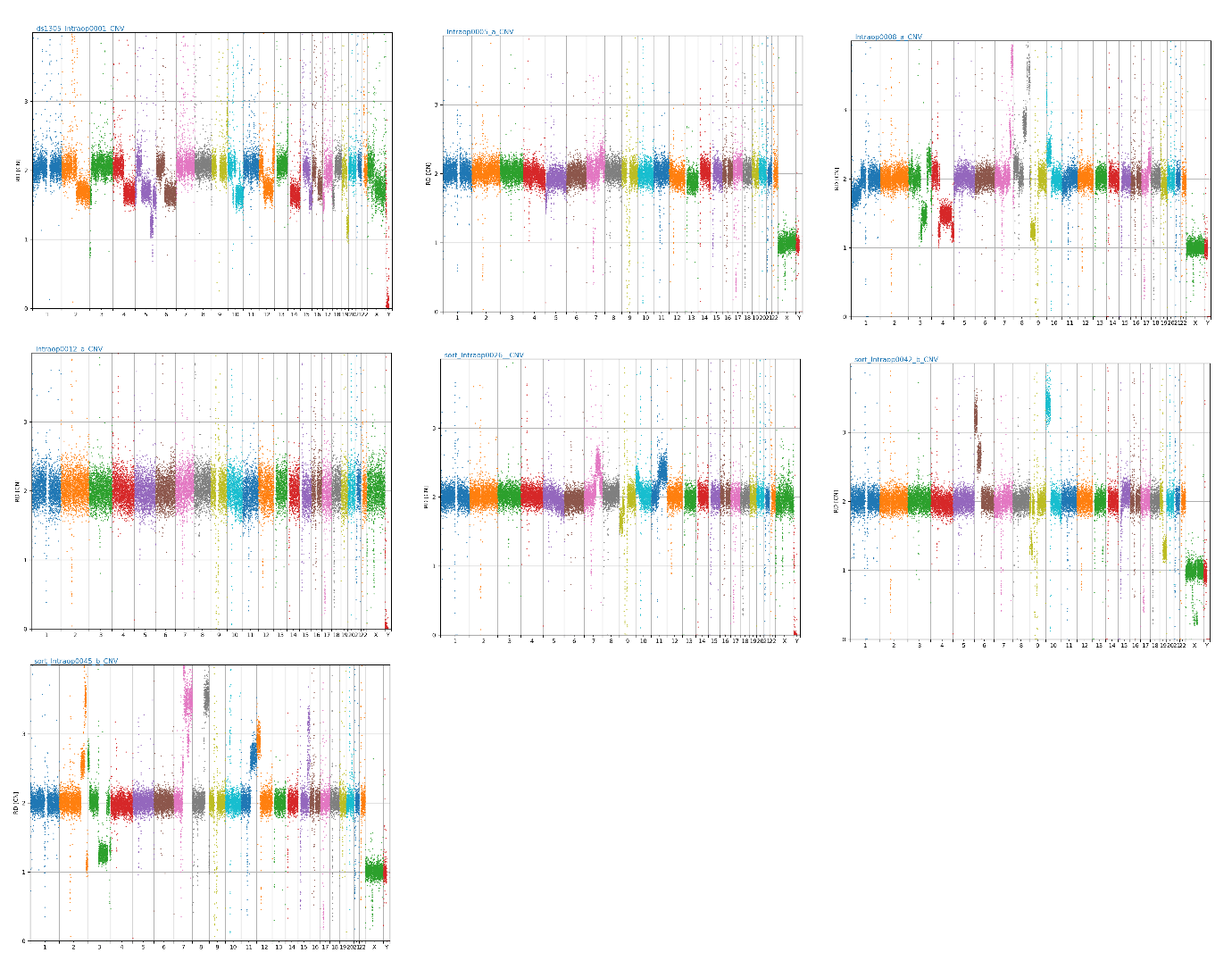
**

**Supplementary Data Figure 2: Copy number plot of all astrocytomas in the prospective cohort**

Note co-deletion of the CDKN2A/B locus (chr9) in cases 8, 26 and 42. These codeletions were confirmed by SoC testing and have prognostic significance (CNS WHO grade 4), despite all cases classifying on methylation array and nanopore as astrocytoma, lower grade.


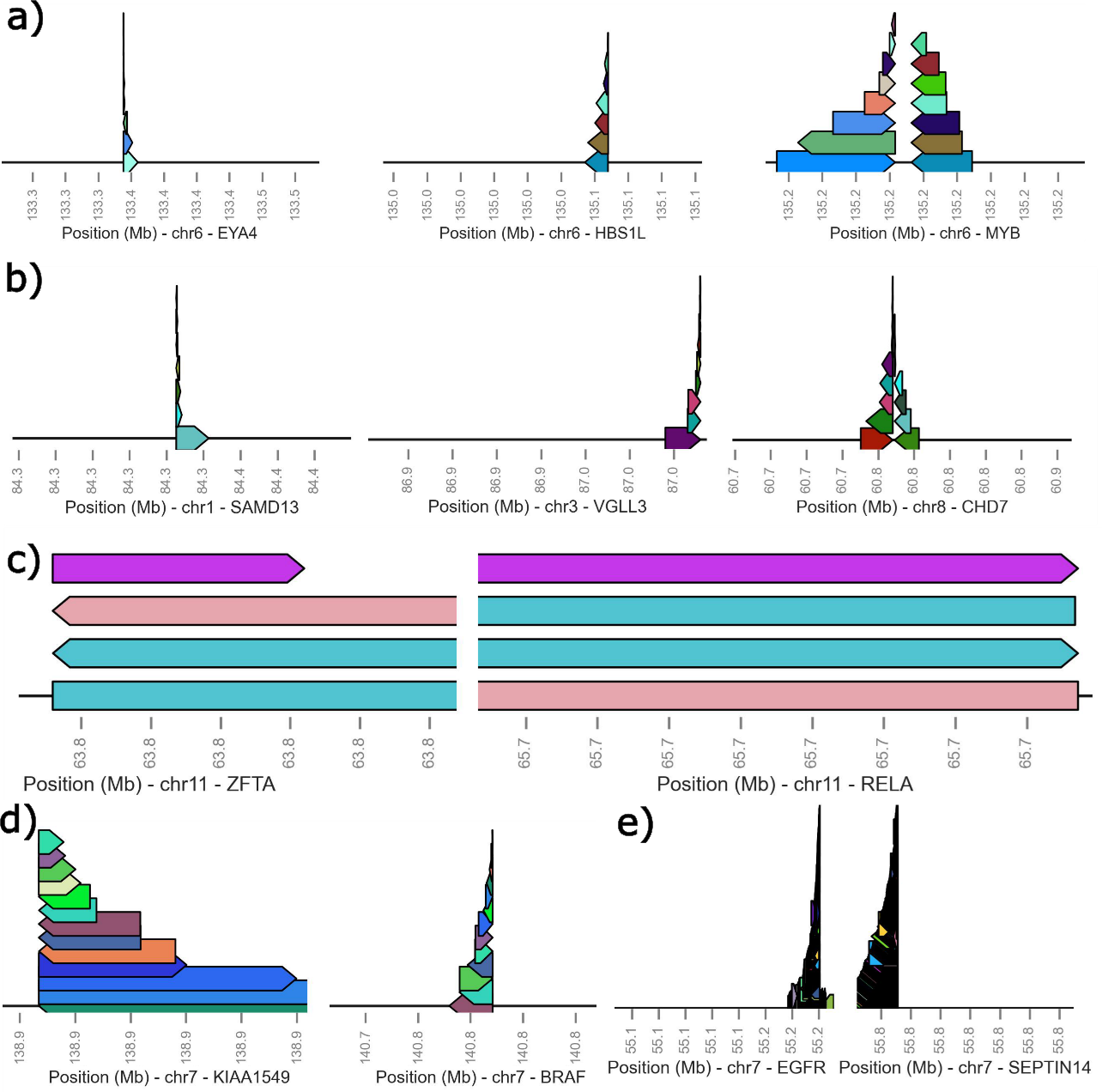


**Supplementary Data Figure 3: Pathognomonic fusion events within the intraoperative cohort.**

Bars each represent a single read, with the colour highlighting the same read mapping across the fusion. A) MYB-altered astrocytoma. B) VGLL-fused intracranial schwannoma. C) ZFTA::RELA fused ependymoma. D) BRAF::KIAA1549 fusion in pilocytic astrocytoma. E) EGFR::SEPTIN14 fused glioblastoma.

**
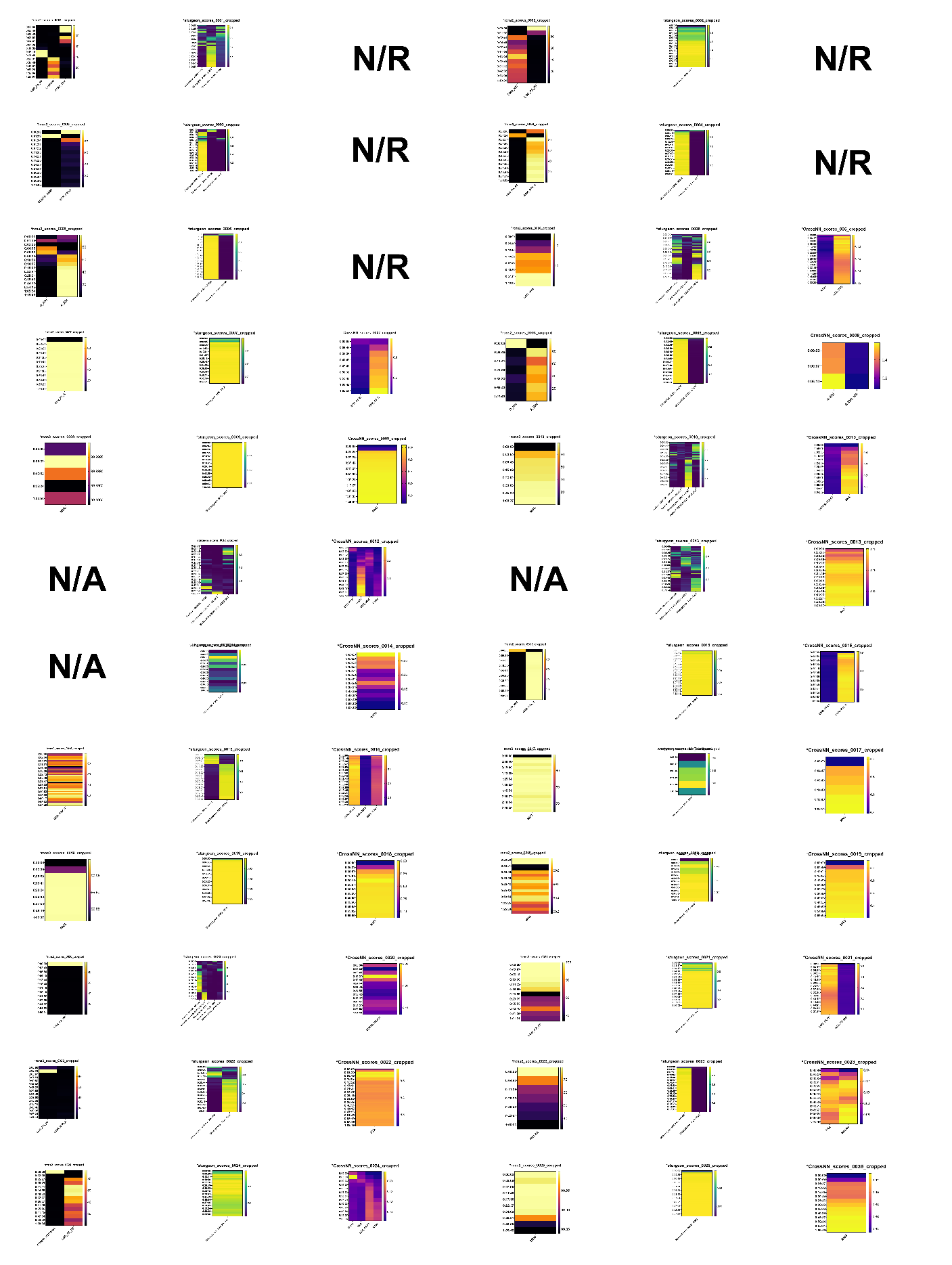
**

**Supplementary Data Figure 4: Intraoperative sequencing results over 1 hour - Cases 1-25**

Random forest subclasses shown where score reached a threshold of >70 at any timepoint. Sturgeon subclasses shown where score reached a threshold of >0.7 at any timepoint. CrossNN subclasses shown where score reached a threshold of >0.1 at any timepoint. CrossNN was not available for intraoperative reporting for the first 5 cases (N/R). N/A indicates where no subclass met threshold during sequencing.

**
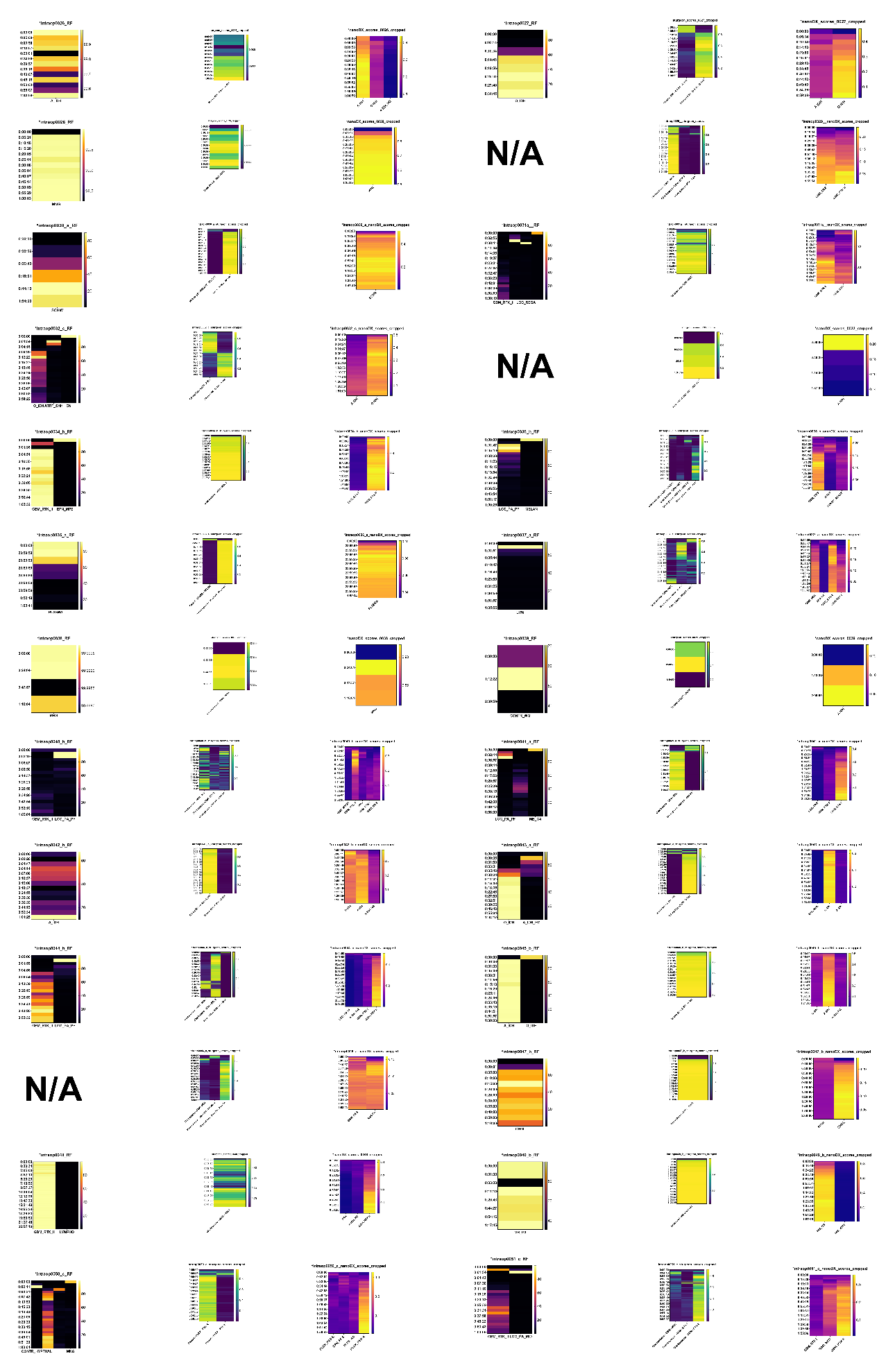
**

**Supplementary Data Figure 4 (cont.): Intraoperative sequencing results over 1 hour - Cases 26-51**

**
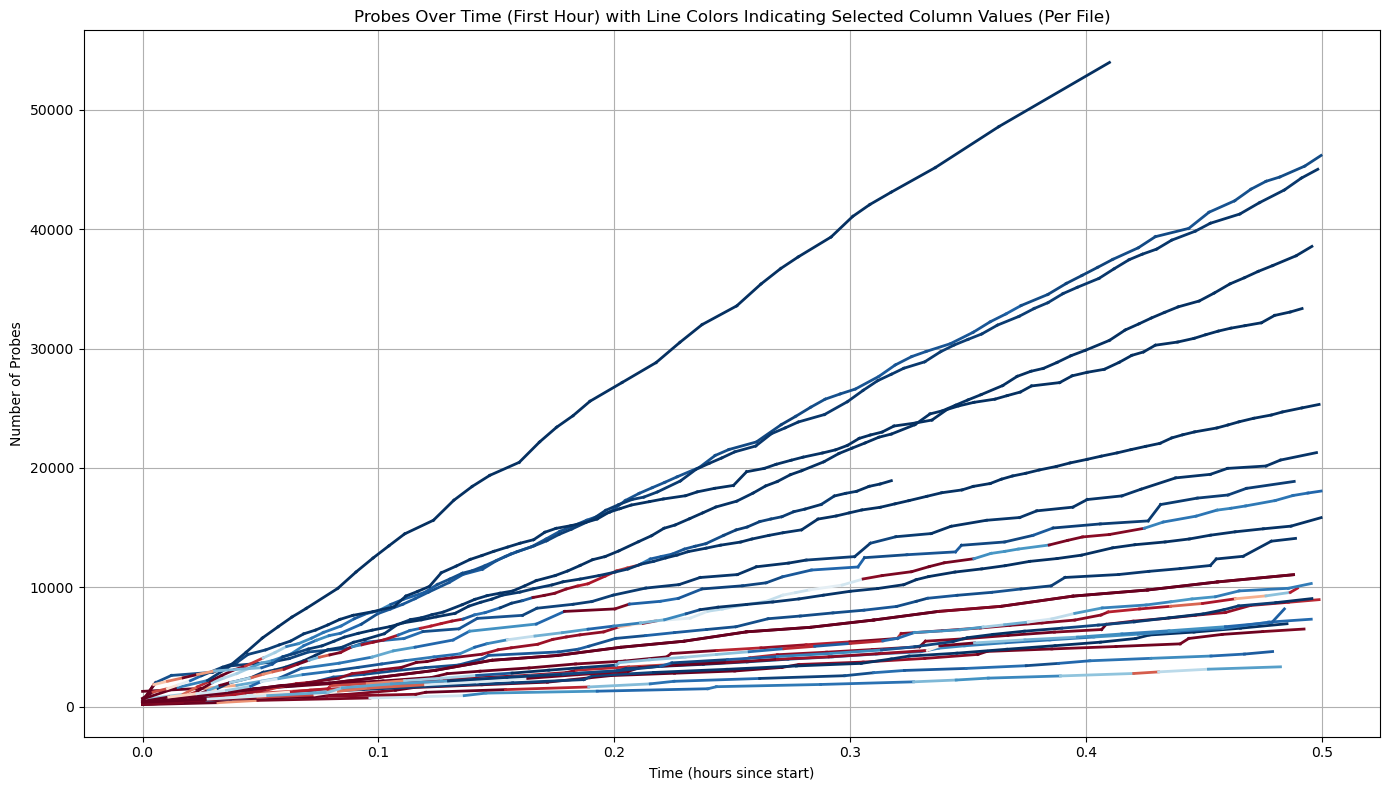
**

**
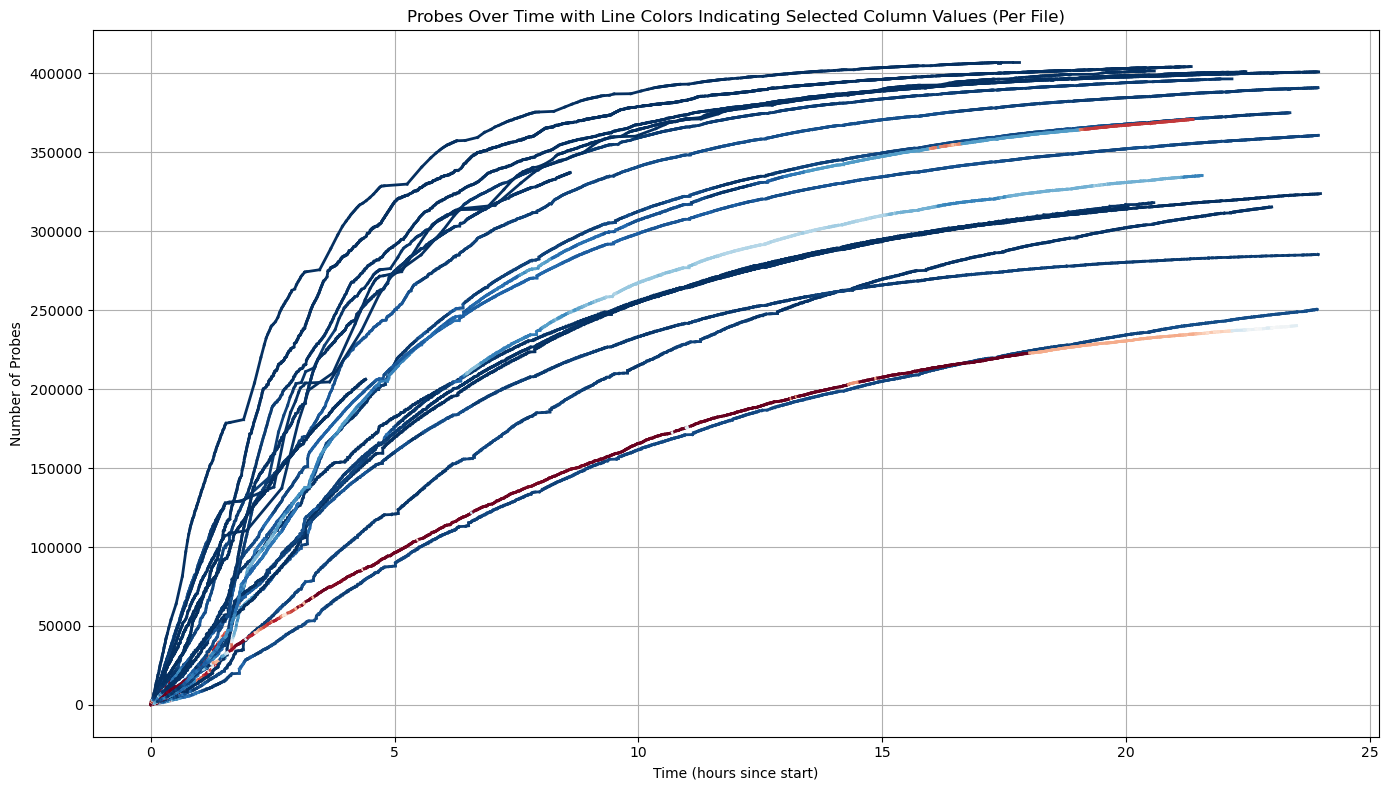
**

**Supplementary Data Figure 5: Number of sites used in sturgeon classifier over time: 30 minutes (top pane) and 24 hours (lower pane).** Lines are coloured according to methylation classification score: lower bound red, upper bound blue. As sequencing runtime increases within the early intraoperative sequencing period, classification scores trend higher (top pane).

| Sample | | RapidCNS2 Subclass | Conf. Score | Sturgeon Subclass | Conf.  Score | crossnn  sUBCLASS | Conf.  Score | Standard of Care Methylation Array | Score | SoC TAT (days) |
| --- | --- | --- | --- | --- | --- | --- | --- | --- | --- | --- |
| 1 | A_IDH | | 100 | Glioma IDH - A IDH - A IDH | 0.9919 | A IDH | 0.44 | Astrocytoma, IDH-mutant, lower grade | 0.99 | 33 |
| 2 | DMG_K27 | | 72 | Glioblastoma - DMG - K27 | 0.9047 | DMG – K27 | 0.33 | Adult-type diffuse high grade glioma, IDH-wildtype, subtypeB | 0.99 | 32 |
| 3 | EPN_RELA | | 54.4 | Ependymal - EPN - RELA | 0.9867 | EPN - RELA | 0.79 | Supratentorial ependymoma, ZFTA fusion-positive | 0.99 | 19 |
| 4 | GBM_RTK_II | | 100 | Glioblastoma - GBM - RTK II | 0.9636 | GBM, RTK II | 0.48 | Glioblastoma, IDH-wildtype, RTK2 subtype | 0.97 | 33 |
| 5 | A_IDH | | 100 | Glioma IDH - A IDH - A IDH | 0.9934 | A IDH | 0.52 | Astrocytoma, IDH-mutant, lower grade | 0.99 | 37 |
| 6 | LGG_MYB | | 97.6 | Other glioma - LGG MYB - MYB | 0.9356 | LGG, MYB | 0.31 | Diffuse astrocytoma, MYB or MYBL1-altered, subtype C | 0.99 | 81 |
| 7 | EPN_PF_A | | 100 | Ependymal - EPN - PF A | 0.9968 | EPN, PF A | 0.84 | Posterior fossa group A ependymoma, subclass 1c | 0.94 | 29 |
| 8 | A_IDH | | 100 | Glioma IDH - A IDH - A IDH | 0.9923 | A IDH | 0.49 | Astrocytoma, IDH-mutant, lower grade | 0.99 | 27 |
| 9 | MNG | | 100 | Mesenchymal - MNG - MNG | 0.9996 | MNG | 0.94 | *Not Requested* | - | - |
| 10 | MNG | | 100 | Mesenchymal - MNG - MNG | 0.9460 | MNG | 0.54 | Meningioma, benign 1 | 0.99 | 42 |
| 12 | CONTR_HEMI | | 5.7 | Glioma IDH - A IDH - A IDH | 0.7563 | A IDH | 0.13 | Astrocytoma, IDH-mutant, lower grade | 0.98 | 39 |
| 13 | PXA | | 28 | Other glioma - PXA - PXA | 0.8117 | PXA | 0.17 | Subependymal Giant Cell Astrocytoma | 0.42 | 46 |
| 14 | A_IDH | | 25.5 | Glioma IDH - O IDH - O IDH | 0.9095 | O IDH | 0.13 | Diffuse Paediatric-type high grade glioma, RTK1, subclass A | 0.64 | 44 |
| 15 | GBM_RTK_II | | 100 | Glioblastoma - GBM - RTK II | 0.9853 | GBM, RTK II | 0.61 | *Not Requested* | - | - |
| 16 | GBM_RTK_II | | 99.4 | Glioblastoma - GBM - RTK II | 0.9149 | GBM, RTK II | 0.33 | *Not Requested* | - | - |
| 17 | MNG | | 100 | Mesenchymal - MNG - MNG | 0.9981 | MNG | 0.74 | Meningioma, benign 3 | 0.87 | 36 |
| 18 | MNG | | 100 | Mesenchymal - MNG - MNG | 0.9996 | MNG | 0.94 | Meningioma, benign 2 | 0.99 | 66 |
| 19 | MNG | | 100 | Mesenchymal - MNG - MNG | 0.9983 | MNG | 0.86 | Meningioma, benign 2 | 0.99 | 31 |
| 20 | CONTR_REACT | | 42.1 | Control - CONTR - INFLAM | 0.7920 | CONTR, REACT | 0.18 | Chordoma | 0.92 | 34 |
| 21 | LGG_PA_PF | | 99.9 | Other glioma - LGG PA - PA | 0.9723 | LGG, PA PF | 0.58 | Infratentorial piloctytic astrocytoma | 0.99 | 27 |
| 22 | GBM_RTK_II | | 66.3 | Other glioma - PXA - PXA | 0.9895 | PXA | 0.46 | Pleomorphic xanthoastrocytoma | 0.99 | 29 |
| 23 | MELAN | | 60.4 | Melanocytic - MELAN - MELAN | 0.9802 | MELAN | 0.20 | No matching methylation class | <0.3 | 32 |
| 24 | LGG_PA_PF | | 77.2 | Glioma IDH - A IDH - A IDH | 0.4802 | LGG, PA PF | 0.19 | Infratentorial piloctytic astrocytoma | 0.51 | 24 |
| 25 | MNG | | 100 | Mesenchymal - MNG - MNG | 0.9998 | MNG | 0.95 | Meningioma, benign 3 | 0.47 | 27 |

**Supplementary Data Table 1: Overview of Intraoperative cases 1-25** *Case 11 excluded as metastasis on smear*)

| Sample | RapidCNS2 Subclass | Conf. Score | Sturgeon Subclass | Conf. Score | cROSSNN  sUBCLASS | cONF.  sCORE | Standard of Care Methylation Array | Score | SOC TAT (days) |
| --- | --- | --- | --- | --- | --- | --- | --- | --- | --- |
| 26 | A_IDH | 100 | Glioma IDH - A IDH - A IDH | 0.9922 | A IDH | 0.50 | Astrocytoma, IDH-mutant; lower grade | 0.99 | 27 |
| 27 | O_IDH | 98.1 | Glioma IDH - O IDH - O IDH | 0.9181 | O IDH | 0.45 | Oligodendroglioma, IDH-mutant and 1p/19q-codeleted | 0.95 | 27 |
| 28 | MNG | 100 | Mesenchymal - MNG - MNG | 0.9995 | MNG | 0.92 | Meningioma, benign 2 | 0.82 | 28 |
| 29 | GBM_RTK_II | 14.8 | Glioblastoma - GBM - MES | 0.9675 | GBM, MES | 0.28 | Glioblastoma, IDH-wildtype, typical mesenchymal type | 0.98 | 36 |
| 30 | SCHW | 90.3 | Nerve - SCHW - SCHW | 0.9586 | SCHW | 0.50 | Schwannoma | 0.52 | 21 |
| 31 | GBM_RTK_II | 69.4 | Glioblastoma - GBM - MES | 0.9453 | GBM, RTK II | 0.41 | Glioblastoma, IDH-wildtype, [typical mesenchymal type] | 0.57 | 32 |
| 32 | O_IDH | 98.5 | Glioma IDH - O IDH - O IDH | 0.9185 | O IDH | 0.49 | Oligodendroglioma, IDH-mutant and 1p/19q-codeleted | 0.94 | 20 |
| 33 | CONTR_HEMI | 7.2 | Glioma IDH - A IDH - A IDH | 0.6111 | CONTR, REACT | 0.08 | Control tissue, white matter (corpus callosum) | 0.93 | 35 |
| 34 | GBM_RTK_II | 100 | Glioblastoma - GBM - RTK II | 0.9180 | GBM, RTK II | 0.50 | Glioblastoma, IDH-wildtype, RTK2 subtype | 0.98 | 18 |
| 35 | PXA | 11.1 | Glioblastoma - GBM - MES | 0.8027 | GBM, MES | 0.28 | Glioblastoma, IDH-wildtype, typical mesenchymal type | 0.99 | 31 |
| 36 | CONTR_REACT | 4.2 | Control - CONTR - INFLAM | 0.9206 | PLASMA | 0.09 | *Not requested (IHC conclusive of Germinoma)* | - | - |
| 37 | GBM_RTK_II | 11.9 | Glioblastoma - GBM - MES | 0.8793 | GBM, RTK II | 0.22 | Glioblastoma, IDH-wildtype, RTK2 subtype | 0.15 | 27 |
| 38 | MNG | 100 | Mesenchymal - MNG - MNG | 0.9997 | MNG | 0.94 | Meningioma, benign 1 | 0.83 | 40 |
| 39 | CONTR_HYPTHAL | 14.8 | Glioma IDH - A IDH - A IDH | 0.7797 | A IDH | 0.06 | control tissue, white matter (corpus callosum) | 0.24 | 42 |
| 40 | GBM_RTK_II | 37.3 | Glioblastoma - GBM - MES | 0.6527 | GBM, RTK I | 0.16 | No matching methylation classes with score >= 0.3 | <0.3 | 34 |
| 41 | GBM_RTK_II | 65 | Glioblastoma - GBM - RTK I | 0.8473 | GBM, RTK I | 0.49 | Glioblastoma, IDH-wildtype, RTK1 subtype | 0.99 | 33 |
| 42 | A_IDH | 99.6 | Glioma IDH - A IDH - A IDH | 0.9877 | A IDH | 0.39 | Astrocytoma, IDH-mutant, lower grade | 0.99 | 33 |
| 43 | O_IDH | 100 | Glioma IDH - O IDH - O IDH | 0.9913 | O IDH | 0.60 | Oligodendroglioma, IDH-mutant and 1p/19q-codeleted | 0.97 | 32 |
| 44 | GBM_RTK_II | 97 | Glioblastoma - GBM - RTK II | 0.9563 | GBM, RTK II | 0.49 | Glioblastoma, IDH-wildtype, RTK2 subtype | 0.98 | 29 |
| 45 | A_IDH | 100 | Glioma IDH - A IDH - A IDH | 0.9928 | A IDH | 0.49 | Astrocytoma, IDH-mutant, lower grade | 0.99 | 29 |
| 46 | LGG_PA_PF | 10 | Other glioma - ANA PA - ANA PA | 0.7444 | GBM, MES | 0.13 | High-grade astrocytoma with piloid features | 0.53 | 27 |
| 47 | CHGL | 71 | Other glioma - CHGL - CHGL | 0.8746 | CHGL | 0.23 | Chordoid glioma, PRKCA mutant | 0.99 | 35 |
| 48 | GBM_RTK_II | 99 | Glioblastoma - GBM - RTK II | 0.8567 | GBM, RTK II | 0.51 | No matching methylation classes with score >= 0.3 | <0.3 | 35 |
| 49 | MB_G3 | 99 | Embryonal - MB G3G4 - G3 | 0.9981 | MB, G3 | 0.82 | Medulloblastoma Group 3, subclass II | 0.99 | 43 |
| 50 | PLEX_PED_A | 77 | Plexus - PLEX - PED A | 0.9935 | PLEX, PED A | 0.39 | Choroid Plexus Papilloma, paediatric subtype | 0.99 | 36 |
| 51 | GBM_RTK_II | 90 | Glioblastoma - GBM - RTK II | 0.8643 | GBM, RTK II | 0.30 | Glioblastoma, RTK1 subtype | 0.79 | 35 |

**Supplementary Data Table 1 Continued: Overview of Intraoperative cases 26-51**

| Sample | RapidCNS2 Subclass | Conf.  Score | Sturgeon Subclass | Conf.  Score | CROSSNN Subclass | ConF  Score | Standard of Care Methylation Array | ConF  Score |
| --- | --- | --- | --- | --- | --- | --- | --- | --- |
| 1 | GBM_RTK_II | 88.5 | Glioblastoma - GBM - MES | 0.929 | GBM, RTK II | 0.291 | Glioblastoma, IDH-wildtype, RTK2 subtype | 0.96 |
| 2 | O_IDH | 55.4 | Glioma IDH - O IDH - O IDH | 0.760 | O IDH | 0.272 | NOT REQUIRED | - |
| 3 | MNG | 100 | Mesenchymal - MNG - MNG | 1.000 | MNG | 0.886 | Meningioma, subtype benign, subclass 3 (novel) | 0.91 |
| 4 | LGG_PA_PF | 35.2 | Sella - PITAD STH - STH DNS B | 0.992 | LGG, PA PF | 0.357 | Pilocytic astrocytoma, infratentorial | 0.99 |
| 5 | GBM_RTK_II | 100 | Glioblastoma - GBM - RTK II | 0.850 | GBM, RTK I | 0.274 | Glioblastoma, IDH-wildtype, RTK 1 subtype | 0.99 |
| 6 | EPN_SPINE | 100 | Ependymal - EPN - SPINE | 0.999 | EPN, SPINE | 0.733 | Ependymoma, spinal | 0.99 |
| 7 | O_IDH | 11.8 | Glio-neuronal - DLGNT - DLGNT | 0.985 | O IDH | 0.103 | Diffuse glioneuronal tumour, subtype A (novel) | 0.99 |
| 8 | A_IDH | 95.1 | Glioma IDH - A IDH - A IDH | 0.991 | A IDH | 0.507 | Astrocytoma, IDH-mutant; lower grade | 0.81 |
| 9 | O_IDH | 15.6 | Glio-neuronal - DLGNT - DLGNT | 0.983 | DMG, K27 | 0.115 | Anaplastic neuroepithelial tumour with condensed nuclei | 0.97 |
| 10 | LGG_PA_PF | 96.4 | Sella - PITAD STH - STH DNS B | 0.994 | LGG, PA PF | 0.365 | Midline pilocytic astrocytoma | 0.87 |
| 11 | GBM_RTK_II | 75.6 | Glioblastoma - GBM - RTK II | 0.874 | GBM, RTK II | 0.396 | No matching methylation classes | <0.3 |
| 12 | GBM_RTK_II | 9.9 | Glioblastoma - GBM - MES | 0.985 | GBM, MES | 0.286 | Glioblastoma, IDH-wildtype, mesenchymal subtype | 0.88 |
| 13 | LGG_PA_GG_ST | 17.8 | Other glioma - LGG PA - PA/GG ST | 0.935 | LGG, PA/GG ST | 0.132 | Pilocytic astrocytoma, hemsipheric | 0.99 |
| 14 | PXA | 87.4 | Other glioma - PXA - PXA | 0.993 | PXA | 0.456 | Glioblastoma, IDH-wildtype, [atypical mesenchymal type] | 0.87 |
| 15 | SUBEPN_PF | 94.7 | Ependymal - SUBEPN - ALL | 0.984 | SUBEPN, PF | 0.594 | Posterior fossa subependymoma | 0.99 |
| 16 | O_IDH | 97.1 | Glioma IDH - O IDH - O IDH | 0.954 | O IDH | 0.282 | Oligodendroglioma, IDH-mutant and 1p/19q-codeleted | 0.96 |
| 17 | A_IDH | 75.3 | Glioma IDH - A IDH - A IDH | 0.911 | A IDH | 0.204 | Astrocytoma, IDH-mutant; lower grade | 0.98 |
| 18 | LGG_DNT | 62.6 | Glio-neuronal - DLGNT - DLGNT | 0.844 | LGG, DNT | 0.203 | Dysembryoplastic neuroepithelial tumour | 0.8 |
| 19 | A_IDH | 99.9 | Glioma IDH - A IDH - A IDH | 0.991 | A IDH | 0.549 | Astrocytoma, IDH-mutant; lower grade | 0.99 |
| 20 | EPN_SPINE | 100 | Ependymal - EPN - SPINE | 0.999 | EPN, SPINE | 0.680 | Spinal ependymoma | 0.99 |
| 21 | MNG | 98.9 | Mesenchymal - MNG - MNG | 0.974 | MNG | 0.411 | Meningioma, subclass benign 3 | 0.96 |
| 22 | LGG_PA_PF | 100 | Other glioma - LGG PA - PA | 0.984 | LGG, PA PF | 0.636 | Infratentorial pilocytic astrocytoma | 0.99 |
| 23 | PLEX_PED_A | 89.9 | Plexus - PLEX - PED A | 0.997 | PLEX, PED A | 0.335 | Choroid plexus papilloma, pediatric subtype | 0.99 |
| 24 | SUBEPN_PF | 68.7 | Ependymal - SUBEPN - ALL | 0.816 | SUBEPN, PF | 0.475 | Subependymoma, posterior fossa | 0.99 |
| 25 | CONTR_REACT | 68.5 | Control - CONTR - REACT | 0.817 | CONTR, REACT | 0.166 | MC control tissue, reactive tumour microenvironment | 0.41 |
| 26 | CONTR_CEBM | 67.7 | Control - CONTR - CEBM | 0.974 | CONTR, CEBM | 0.256 | MC High-grade astrocytoma with piloid features | 0.94 |
| 27 | A_IDH | 98.4 | Glioma IDH - A IDH - A IDH | 0.986 | A IDH | 0.503 | MC Astrocytoma, IDH-mutant; lower grade | 0.94 |
| 28 | GBM_G34 | 97.3 | Glioblastoma - GBM - G34 | 0.991 | GBM, G34 | 0.655 | Diffuse hemispheric glioma, H3 G34-mutant | 0.99 |
| 29 | GBM_RTK_II | 78.6 | Glioblastoma - GBM - RTK II | 0.878 | GBM, RTK II | 0.291 | Diffuse paediatric-type high grade glioma, RTK2 subtype, subclass B (novel) | 0.28 |
| 30 | GBM_RTK_II | 99.8 | Glioblastoma - GBM - RTK II | 0.961 | GBM, RTK II | 0.465 | Glioblastoma, IDH-wildtype, RTK2 subtype | 0.35 |

**Supplementary Data Table 2: Overview of Retrospective cases**

| Sample | Nanopore Subclass | Array Subclass | V12_Score | Final Integrated Diagnosis | Notes |
| --- | --- | --- | --- | --- | --- |
| 11 | GBM, RTK II | No matching methylation classes | <0.3 | Glioblastoma, IDH-wildtype, CNS WHO grade 4 | Unclassifiable |
| 14 | PXA | Glioblastoma, IDH-wildtype, [atypical mesenchymal type] | 0.87 | Epithelioid glioblastoma, CNS WHO grade 4, BRAF mutant | Large overlap between PXA and epithelioid GBM |
| 25 | CONTROL REACT | MC control tissue, reactive tumour microenvironment | 0.41 | Diffuse midline glioma, H3 K27-altered, CNS WHO grade 4 | Poor tissue selection |

**Supplementary Data Table 3a: Discrepant cases in retrospective cohort**

| Sample | Nanopore Subclass | Array Subclass | V12_Score | Final Integrated Diagnosis | Notes |
| --- | --- | --- | --- | --- | --- |
| 9 | O_IDH *vs* DMG, K27 vs DLGNT | Anaplastic neuroepithelial tumour with condensed nuclei (novel) | 0.97 | High-grade neuroepithelial tumour, NTRK2 fusion-positive | Novel entity (not included in v11) |
| 26 | CONTROL CEBM | MC High-grade astrocytoma with piloid features | 0.94 | High-grade astrocytoma with piloid features, CNS WHO grade 3 | Novel entity (not included in v11); Poor tissue selection |
| 29 | GBM, RTK II | Diffuse paediatric-type high grade glioma, RTK2 subtype, subclass B (novel) | 0.28 | Glioblastoma, IDH-wildtype, CNS WHO grade 4 (Chr7-/10+) | Novel entity (not included in v11 (low score) |

**Supplementary Data Table 3b: Novel cases in retrospective cohort**

| Sample | Nanopore Subclass | Array Subclass | V12_Score | Final Integrated Diagnosis | Notes |
| --- | --- | --- | --- | --- | --- |
| 12 | CONTROL *vs* A_IDH | Astrocytoma, IDH-mutant, lower grade | 0.98 | Astrocytoma, IDH-mutant, CNS WHO 2 | Low tumour DNA fraction |
| 20 | CONTR_REACT *vs* CHORDOMA* | Chordoma | 0.92 | Conventional Chordoma | Poor tissue selection |
| 22 | GBM_RTK_II *vs* PXA | Pleomorphic xanthoastrocytoma | 0.99 | Pleomorphic xanthoastrocytoma, CNS WHO 3 | Necrosis & microvascular proliferation |
| 24 | LGG_PA_PF *vs* A IDH | Infratentorial piloctytic astrocytoma | 0.51 | Piloctytic astrocytoma, CNS WHO 1 | Low array score |
| 33 | CONTR_HEMI *vs* A IDH | Control tissue, white matter (corpus callosum) | 0.93 | CNS tissue fragments with diffuse infiltration of a glioma | Low tumour DNA purity; post-radiotherapy |
| 35 | PXA *vs* GBM MES | Glioblastoma, IDH-wildtype, typical 0.99  mesenchymal type | | Glioblastoma, CNS WHO 4 |  |
| 39 | CONTR_HYPTHAL *vs* A_IDH | control tissue, white matter (corpus callosum) | 0.24 | CNS white matter with focal tumour infiltrates and microvascular proliferation | Low tumour DNA purity |

**Supplementary Data Table 4a: Discrepant cases in intraoperative cohort**

*Sample 20 was discrepant over time during intraoperative sequencing, oscillating between chordoma and control tissue classifications

| Sample | Nanopore Subclass | Array Subclass | V12_Score | Final Integrated Diagnosis | Notes |
| --- | --- | --- | --- | --- | --- |
| 2 | DMG_K27 | Adult-type diffuse high-grade glioma, IDH-wildtype, subtype B | 0.99 | Adult-type diffuse high grade glioma, IDH-wildtype, subtype B, WHO 4 | Novel entity (not included in v11) |
| 13 | PXA | Subependymal Giant Cell Astrocytoma | 0.42 | Glial Neoplasm, NOS | Unclassifiable; Low array score |
| 14 | A_IDH *vs* O IDH | Diffuse Paediatric-type high grade glioma, RTK1, subclass A | 0.64 | Suggestive of Diffuse Paediatric-type high grade glioma, H3 wildtype IDH wildtype CNS WHO 4 | Novel entity (not included in v11); Adult Patient; Low array score |
| 36 | CONTR_REACT *vs* *Not performed*  CONTR_INFLAM | |  | Germinoma | Novel entity (not included in v11) |
| 46 | LGG_PA_PF *vs* ANA PA | High-grade astrocytoma with piloid features | 0.53 | High grade glioma, methylation profile high grade astrocytoma with piloid features (best corresponding to CNS WHO grade 3) | Novel entity (not included in v11); Low array score |

**Supplementary Data Table 4b: Novel entities in intraoperative cohort**

| Astrocytoma | | | | |
| --- | --- | --- | --- | --- |
| Case | IDH1 Status | TP53 Status | 1p/19q | CKDN2A/B |
| 1 | p.R132H | p.R81Q | No | Intact |
| 5 | p.R132C | p.R141L | No | Intact |
| 8 | p.R132H | p.R141C | No | Del |
| 12* | *Not found* | *Not found* | No | Intact |
| 26 | p.R132H | p.R141H | No | Del |
| 42 | p.R132H | p.R116W | No | Del |
| 45 | p.R132H | p.A29T | No | Intact |

*Low tumour DNA purity (classified as control tissue)

| Glioblastoma | | | | |
| --- | --- | --- | --- | --- |
| Case | IDH mutation | TERT | TP53 | gain 7/ loss 10 |
| 4 | No | Upstream | p.V65L | Yes (& complex) |
| 15 | No | Upstream | *Not found* | Yes (& complex) |
| 16 | No | Upstream | p.E153K | Yes (& complex) |
| 29 | No | *Not found* | p.R210P | Yes (& complex) |
| 31 | No | Upstream | p.R210Q | Yes |
| 34 | No | Upstream | *Not found* | Yes |
| 35 | No | *Not found* | *Not found* | Yes |
| 37 | No | Upstream | *Not found* | Yes |
| 40 | No | Upstream | *Not found* | Yes (& complex) |
| 41 | No | *Not found* | *Not found* | Yes (& complex) |
| 44 | No | *Not found* | *Not found* | Yes |
| 48 | No | Upstream | *Not found* | Yes |

| Oligodendroglioma | | | | |
| --- | --- | --- | --- | --- |
| Case | IDH Status | ATRX Status | TERT | 1p/19q codeletion |
| 27 | IDH2 p.R42K | Intact | Upstream | Yes |
| 32 | IDH1 p.R132H | Intact | *Not found* | Yes |
| 43 | IDH1 p.R132H | Intact | Upstream | Yes |

**Supplementary Data Table 5: Summary of selected additional diagnostic information**: *Top pane:* Astrocytoma. *Middle pane*: Glioblastoma. *Lower Pane*: Oligodendroglioma.

|  | Nanopore | | | Array | | | |
| --- | --- | --- | --- | --- | --- | --- | --- |
| Case | Coverage (x) | Methylation (%) | Status (Cutoff = 25%) | Status (Cutoff = 0.3582) | Methylation | Lower CI | Upper CI |
| 1 | 23 | 47.49 | methylated | methylated | 0.9834999 | 0.8424044 | 0.998498 |
| 2 | 31 | 4.99 | unmethylated | unmethylated | 0.0832796 | 0.0189851 | 0.2989573 |
| 3 | 13 | 13.93 | unmethylated | unmethylated | 0.0799639 | 0.0216062 | 0.254882 |
| 4 | 18 | 26.43 | unmethylated | unmethylated | 0.1479952 | 0.0402688 | 0.418302 |
| 5 | 27 | 46 | methylated | methylated | 0.9691314 | 0.8125346 | 0.995622 |
| 6 | 19 | 7.32 | unmethylated | unmethylated | 0.0288208 | 0.0048703 | 0.152502 |
| 7 | 3 | N/A | N/A | unmethylated | 0.0617912 | 0.0128008 | 0.250666 |
| 8 | 35 | 41.38 | methylated | methylated | 0.9257303 | 0.6956579 | 0.985501 |
| 9 | 27 | 7.42 | unmethylated | *Not performed* |  |  |  |
| 10 | 14 | 8.76 | unmethylated | unmethylated | 0.0389551 | 0.0075663 | 0.177297 |
| 12* | 8 | 10.15 | **unmethylated** | **methylated** | 0.6913067 | 0.3825578 | 0.890043 |
| 13 | 11 | 9.49 | unmethylated | unmethylated | 0.0922101 | 0.0264277 | 0.275413 |
| 14 | 29 | 76.21 | methylated | methylated | 0.9967815 | 0.9385104 | 0.999841 |
| 15 | 7 | 60.41 | methylated | *Not performed* |  |  |  |
| 16 | 32 | 60.19 | methylated | *Not performed* |  |  |  |
| 17 | 22 | 6.36 | unmethylated | unmethylated | 0.0543459 | 0.0125038 | 0.206873 |
| 18 | 10 | 6.32 | unmethylated | unmethylated | 0.0289705 | 0.0048849 | 0.1534933 |
| 19 | 14 | 6.94 | unmethylated | unmethylated | 0.0548605 | 0.0127066 | 0.207472 |
| 20 | 20 | 4.84 | unmethylated | unmethylated | 0.047018 | 0.0101 | 0.192622 |
| 21 | 19 | 16.35 | unmethylated | unmethylated | 0.0437162 | 0.0089011 | 0.188769 |
| 22 | 5 | 5.83 | unmethylated | unmethylated | 0.089189 | 0.0244349 | 0.276848 |
| 23 | 9 | 4.21 | unmethylated | unmethylated | 0.0097327 | 0.0010217 | 0.0863 |
| 24 | 18 | 13.63 | unmethylated | unmethylated | 0.0378514 | 0.007396 | 0.171987 |
| 25 | 28 | 11.2 | unmethylated | unmethylated | 0.0237046 | 0.0038069 | 0.13365 |
| 26 | 31 | 31.71 | methylated | methylated | 0.4706067 | 0.0845057 | 0.8954095 |
| 27 | 15 | 19.9 | unmethylated | unmethylated | 0.1315761 | 0.0396036 | 0.357608 |
| 28 | 17 | 13.09 | unmethylated | unmethylated | 0.0982246 | 0.0272641 | 0.297407 |
| 29 | 8 | 10.47 | unmethylated | unmethylated | 0.0172385 | 0.0024562 | 0.1110812 |
| 30 | 25 | 6.89 | unmethylated | unmethylated | 0.0502665 | 0.0110369 | 0.200644 |
| 31 | 4 | N/A | N/A | unmethylated | 0.0915101 | 0.0249663 | 0.2837928 |
| 32 | 19 | 56.19 | methylated | methylated | 0.9971626 | 0.9477443 | 0.999853 |
| 33 | 13 | 21.42 | unmethylated | unmethylated | 0.313453 | 0.1423486 | 0.5567218 |
| 34 | 9 | 19.03 | unmethylated | unmethylated | 0.0553101 | 0.0121695 | 0.2176825 |
| 35 | 8 | 12.13 | unmethylated | unmethylated | 0.0315812 | 0.0058135 | 0.153884 |
| 36 | 10 | 5.12 | unmethylated | *Not performed* |  |  |  |
| 37 | 20 | 5.96 | unmethylated | unmethylated | 0.0333289 | 0.0062487 | 0.15899 |
| 38 | 24 | 13.78 | unmethylated | unmethylated | 0.0185204 | 0.0025313 | 0.123046 |
| 39 | 7 | 17.88 | unmethylated | unmethylated | 0.0682699 | 0.0162367 | 0.245448 |
| 40 | 7 | 8.42 | unmethylated | unmethylated | 0.0152806 | 0.0020423 | 0.105279 |
| 41 | 6 | 19.43 | unmethylated | unmethylated | 0.0197028 | 0.0029762 | 0.119197 |
| 42 | 30 | 42.53 | methylated | methylated | 0.9346444 | 0.7420585 | 0.986129 |
| 43 | 30 | 72.2 | methylated | methylated | 0.9397614 | 0.614964 | 0.99348 |
| 44 | 11 | 22.43 | **unmethylated** | **methylated** | 0.9016779 | 0.4524042 | 0.990272 |
| 45 | 23 | 35.74 | methylated | methylated | 0.9816494 | 0.8421308 | 0.998139 |
| 46 | 26 | 15.06 | unmethylated | unmethylated | 0.3493738 | 0.1657983 | 0.591971 |
| 47 | 24 | 9.41 | unmethylated | unmethylated | 0.0786054 | 0.0198676 | 0.264191 |
| 48 | 6 | 51.63 | methylated | methylated | 0.9839353 | 0.8142391 | 0.998833 |
| 49 | 9 | 8.99 | unmethylated | unmethylated | *Not provided* |  |  |
| 50 | 5 | 10.43 | unmethylated | unmethylated | 0.2448144 | 0.1004793 | 0.4847506 |
| 51 | 7 | 28.17 | methylated | methylated | 0.9706608 | 0.8207524 | 0.9958341 |

**Supplementary Data Table 6a: MGMT promoter methylation (intraoperative cohort)**

*Case 12 suffered from low tumoral DNA fraction

|  | Nanopore | | EPIC Array |
| --- | --- | --- | --- |
| Sample | Methylation | Status (Cutoff = 25%) | MGMT Status (EPIC) |
| DS1263_1_M1 | 43.12 | methylated | Methylated |
| DS1263_3_M1 | 23.31 | unmethylated | **No array result** |
| DS1263_4_M1 | 9.78 | unmethylated | Unmethylated |
| NUH_CNS_1 | 10.16 | unmethylated | Unmethylated |
| NUH_CNS_2 | 7.33 | unmethylated | Unmethylated |
| NUH_CNS_3 | 12.32 | **unmethylated** | Methylated |
| NUH_CNS_4 | 10.24 | unmethylated | Unmethylated |
| NUH_CNS_5 | 38.97 | methylated | Methylated |
| NUH_CNS_6 | 56.16 | methylated | Methylated |
| NUH_CNS_7 | 9.47 | unmethylated | Unmethylated |
| NUH_CNS_8 | NA | **NA** | Unmethylated |
| NUH_CNS_9 | 29.14 | methylated | Methylated |
| NUH_CNS_10 | NA | **NA** | Unmethylated |
| NUH_CNS_11 | 8.69 | unmethylated | Unmethylated |
| NUH_CNS_12 | 18.36 | unmethylated | Unmethylated |
| NUH_CNS_13 | 34.48 | methylated | Methylated |
| NUH_CNS_14 | 35.72 | methylated | Methylated |
| NUH_CNS_15 | 8.98 | unmethylated | Unmethylated |
| NUH_CNS_16 | 20.42 | **unmethylated** | Methylated |
| NUH_CNS_17 | 15.07 | unmethylated | Unmethylated |
| NUH_CNS_18 | 5.16 | unmethylated | Unmethylated |
| NUH_CNS_19 | 6.23 | unmethylated | Unmethylated |
| NUH_CNS_21 | 9.46 | unmethylated | Unmethylated |
| NUH_CNS_22 | 8.19 | unmethylated | Unmethylated |
| NUH_CNS_23 | 8.35 | unmethylated | Unmethylated |
| NUH_CNS_24 | 34.56 | methylated | Methylated |
| NUH_CNS_25 | 32.58 | methylated | Methylated |
| NUH_CNS_26 | 45.48 | methylated | Methylated |
| NUH_CNS_29 | 58.48 | methylated | Methylated |
| NUH_CNS_30 | 24.85 | unmethylated | Unmethylated |

**Supplementary Data Table 6b: MGMT promoter methylation (retrospective cohort)**

**References**

1. Patel A, Dogan H, Payne A, et al. Rapid-CNS2: rapid comprehensive adaptive nanopore-sequencing of CNS tumors, a proof-of-concept study. Acta Neuropathol. 2022;143(5):609–12.

2. Payne A, Holmes N, Clarke T, Munro R, Debebe BJ, Loose M. Readfish enables targeted nanopore sequencing of gigabase-sized genomes. Nat Biotechnol 2021;39(4):442–50.

3. Smolka M, Paulin LF, Grochowski CM, et al. Comprehensive Structural Variant Detection: From Mosaic to Population-Level. bioRxiv [Internet] 2023; Available from: https://doi.org/10.1101/2022.04.04.487055

4. Scheinin I, Sie D, Bengtsson H, et al. DNA copy number analysis of fresh and formalin-fixed specimens by shallow whole-genome sequencing with identification and exclusion of problematic regions in the genome assembly. Genome Res 2014;24(12):2022–32.

5. Zheng Z, Li S, Su J, Leung AW-S, Lam T-W, Luo R. Symphonizing pileup and full-alignment for deep learning-based long-read variant calling. Nat Comput Sci [Internet] 2022;2(12):797–803. Available from: https://doi.org/10.1038/s43588-022-00387-x

6. Kuschel LP, Hench J, Frank S, et al. Robust methylation-based classification of brain tumors using nanopore sequencing. Neuropathol Appl Neurobiol [Internet] 2023;49(1). Available from: https://doi.org/10.1101/2021.03.06.21252627

7. Yuan D, Jugas R, Pokorna P, et al. crossNN: an explainable framework for cross-platform DNA methylation-based classification of cancer. medrxiv [Internet] 2024;Available from: https://doi.org/10.1101/2024.01.22.24301523

8. Vermeulen C, Pagès-Gallego M, Kester L, et al. Ultra-fast deep-learned CNS tumour classification during surgery. Nature 2023;622(7984):842–9.

9. Capper D, Jones DTW, Sill M, et al. DNA methylation-based classification of central nervous system tumours. Nature 2018;555(7697):469–74.

10. Maas SLN, Stichel D, Hielscher T, et al. Integrated Molecular-Morphologic Meningioma Classification: A Multicenter Retrospective Analysis, Retrospectively and Prospectively Validated. J Clin Oncol [Internet] 2021;39:3839–52. Available from: https://doi.

11. Hielscher T, Sill M, Sievers P, et al. Clinical implementation of integrated molecular-morphologic risk prediction for meningioma. Brain Pathology 2023;33(3).
